# Comparative transcriptome analysis reveals candidate genes related to cadmium accumulation and tolerance in two almond mushroom (*Agaricus brasiliensis*) strains with contrasting cadmium tolerance

**DOI:** 10.1371/journal.pone.0239617

**Published:** 2020-09-29

**Authors:** Peng-Hu Liu, Zai-Xing Huang, Xu-Hui Luo, Hua Chen, Bo-Qi Weng, Yi-Xiang Wang, Li-Song Chen

**Affiliations:** 1 Institute of Plant Nutritional Physiology and Molecular Biology, College of Resources and Environment, Fujian Agriculture and Forestry University, Fuzhou, Fujian, China; 2 National Engineering Research Center of JUNCAO Technology, Fujian Agriculture and Forestry University, Fuzhou, Fujian, China; 3 Fujian Key Laboratory of Agricultural Ecological Process in Red Soil Hilly Region, Agricultural Ecology Institute, Fujian Academy of Agricultural Sciences, Fuzhou, Fujian, China; Tianjin University, CHINA

## Abstract

Cadmium (Cd) is a toxic metal occurring in the environment naturally. Almond mushroom (*Agaricus brasiliensis*) is a well-known cultivated edible and medicinal mushroom. In the past few decades, Cd accumulation in *A*.*brasiliensis* has received increasing attention. However, the molecular mechanisms of Cd-accumulation in *A*. *brasiliensis* are still unclear. In this paper, a comparative transcriptome of two *A*.*brasiliensis* strains with contrasting Cd accumulation and tolerance was performed to identify Cd-responsive genes possibly responsible for low Cd-accumulation and high Cd-tolerance. Using low Cd-accumulating and Cd-tolerant (J77) and high Cd-accumulating and Cd-sensitive (J1) *A*.*brasiliensis* strains, we investigated 0, 2 and 5 mg L^-1^ Cd-effects on mycelium growth, Cd-accumulation and transcriptome revealed by RNA-Seq. A total of 57,884 unigenes were obtained. Far less Cd-responsive genes were identified in J77 mycelia than those in J1 mycelia (e.g., *ABC transporters*, *ZIP Zn transporter*, Glutathione S-transferase and *Cation efflux (CE) family*). The higher Cd-accumulation in J1 mycelia might be due to Cd-induced upregulation of *ZIP Zn transporter*. Cd impaired cell wall, cell cycle, DNA replication and repair, thus decreasing J1 mycelium growth. Cd-stimulated production of sulfur-containing compounds, polysaccharides, organic acids, trehalose, ATP and NADPH, and sequestration of Cd might be adaptive responses of J1 mycelia to the increased Cd-accumulation. DNA replication and repair had better stability under 2 mg L^-1^ Cd, but greater positive modifications under 5 mg L^-1^ Cd. Better stability of DNA replication and repair, better cell wall and cell cycle stability might account for the higher Cd-tolerance of J77 mycelia. Our findings provide a comprehensive set of DEGs influenced by Cd stress; and shed light on molecular mechanism of *A*.*brasiliensis* Cd accumulation and Cd tolerance.

## Introduction

Almond mushroom (*Agaricus brasiliensi*s), one of the important cultivated edible mushrooms and natural foods, has been produced on an industrial scale in Brazil, China and Japan [1–2]. Fruiting body and mycelia cultivated in liquid medium contains a variety of chemical compounds such as polysaccharides, β-glucans, agarol, phenolics, and sterols which have been proved to play a role in immunoregulation, antitumor, hepatoprotection, anti-diabetes, antioxidant and antimicrobial activities, and the prevention of hyperlipidemia and arteriosclerosis [3–9]. Cadmium (Cd), one of a nonessential and natural element, is potentially hazardous to animal and human health. High concentration of Cd, up to 100–300 mg kg^-1^ dry matter (DM) was observed in the genus *Agaricus* [10]. Cd concentrations in *A*. *brasiliensis* ranged from 3–30 mg kg^-1^ DM, which was higher than that in many edible mushroom species [11]. Therefore, Cd accumulation in *A*. *brasiliensis* could potentially affect food safety and eventually have a direct or indirect threat to human health [12]. Over the past decades, Cd-accumulation in *A*. *brasiliensis* has received increasing concerns.

Considerable effort has been invested into investigating Cd stress in *A*. *brasiliensis*, physiological response, Cd-induced genes, and so on. Xu et al. (2011) reported that Cd absorption coefficient of *A*. *brasiliensis* ranged from 65 to 108 indicated it had high absorption ability to Cd, and different strains showed contrasting Cd accumulation and tolerance [13]. Cd concentration in *A*. *brasiliensis* fruiting bodies decreased with increasing yield or fruiting body number, while increased with increasing substrate Cd (phosphorus) concentration or fruiting body size [11]. Using suppression subtractive hybridization combined with mirror orientation selection, Wang et al. [14] identified 39 Cd-induced genes from *A*. *brasiliensis* mycelia and 26 genes displayed significant similarity to known genes. These genes were related to (*a*) metabolism, (*b*) cell rescue, defense and virulence, (*c*) protein fate, (*d*) cellular transport, transport facilitation and transport routes, (*e*) transcription, and (*f*) the action of proteins with a binding function. However, knowledge regarding the physiological and molecular mechanisms of Cd-accumulation in *A*. *brasiliensis* remains very limited.

Screening and breeding low-Cd-accumulation cultivars is a low-cost and high-performance approach to reduce Cd uptake by human *via* food chain [15]. Recently, we bred and identified several strains of *A*. *brasiliensis* having different capacities of Cd-accumulation and Cd-tolerance, and obtaining a low Cd-accumulating strain (J77, Cd-tolerance) and a high Cd-accumulating strain (J1, Cd-sensitivity) in both fruit bodies and mycelia [16, 17]. Understanding the molecular mechanisms underlying low Cd-accumulation is crucial, not only to allow us to screen low-Cd-accumulation *A*. *brasiliensis* cultivars, but also to provide us opportunities to breed Cd pollution-safe *A*. *brasiliensis* cultivars. Recently, high-throughput RNA sequencing (RNA-seq) approach has become increasingly popular in transcriptomics studies. Gene expression profiles revealed by RNA-Seq allow us to discover and characterize genes, and identify and quantify known and/or novel genes massively and simultaneously. To date, comparative transcriptome based on RNA-Seq has been used to investigate the molecular mechanisms of Cd-accumulation and Cd-tolerance in some fungi including *Paxillus involutus* [18], *Exophiala pisciphila* [19], and *Blastocladiella emersonii* [20]. Using this method, many genes that are responsible for the Cd-accumulation and Cd-tolerance has been identified in the above fungi. To our knowledge, such data is very limited in *A*. *brasiliensis*.

In this study, a comparative transcriptome based on RNA-Seq was performed for the mycelia of a low Cd-accumulating and Cd-tolerant strain (J77) bred by irradiating J1 using ^60^Co-γ-ray and a high Cd-accumulating and Cd-sensitive *A*. *brasiliensis* strain (J1) cultured under Cd-stress. The objectives were to reveal the mechanisms underlying low Cd-accumulation and high Cd-tolerance in *A*. *brasiliensis* strain (J77) at the transcriptional level and to identify Cd-responsive genes possibly responsible for low Cd-accumulation and high Cd-tolerance in mycelia.

## Materials and methods

### Sources of *A*. *brasiliensis* strains and Cd treatments

Two *A*. *brasiliensis* strains (low Cd-accumulating, Cd-tolerant J77 and high Cd-accumulating, Cd-sensitive J1) were stored in National Engineering Research Center of JUNCAO Technology, Fujian Agriculture and Forestry University, Fuzhou, China. The two strains were routinely grown on potato dextrose agar in 1 L culture medium supplemented with 2 g KH_2_PO_4_, 0.5 g Mg_2_SO_4_·7H_2_O and 10 mg vitamin B_1_, and then transferred to new culture medium every three months. J77 and J1 mycelia were cultured in the above culture medium at a Cd^2+^ concentration of 0 (Cd0), 2 (Cd2) or 5 (Cd5) mg L^-1^by using CdCl_2_·2.5H_2_O for 25 days. There were three biological replicates per treatment. Thereafter, parts of mycelia from each treatment were collected, immediately frozen in liquid nitrogen, and then stored at -80 °C until they were used for RNA-Seq and qRT-PCR analysis. The unsampled mycelia were used to assay Cd.

### Determination of Cd concentration in mycelium

Mycelium Cd concentration was assayed using a flame atomic absorption spectrophotometer (FAAS, Hitachi Z-2300, Japan) after samples were dried to a constant weight at 70 °C. There were three replicates per treatment.

### RNA extraction, cDNA library construction and Illumina sequencing

Total RNA were extracted from Cd0-, Cd2- and Cd5-treated mycelia of J77 and J1 strains using Recalcitrant Plant Total RNA Extraction Kit (Centrifugal column type, Bioteke, China) following the manufacturer’s instructions, and then treated with RNase-free DNAse I (TaKaRaBiotech Co., Ltd., Dalian, China) to remove residual DNA. There were three biological replicates per treatment. The integrity and quality of total RNA were checked by 1% (w/v) agarose gel electrophoresis and spectrophotometer at 260 and 280 nm. Only RNA samples that had a 260 nm/280 nm absorbance ratio of between 1.8 and 2.0 were used for subsequent analyses.

High-quality RNA samples were sent to Biomarker Technologies Corporation (Beijing, China) for cDNA library construction and sequencing. Magneticoligo (dT) beads were used to enrich the poly (A) mRNA tails of four independent RNA. The enriched mRNA was fragmented into small pieces, which were prepared as templates for cDNA synthesis. Double-stranded cDNA was synthesized using SuperScript II buffer, dNTPs, RNase H, and DNA polymerase I. The yielding cDNA was purified using a QiaQuick PCR extraction kit (Qiagen, Inc., Hilden, Germany) and was eluted with EB buffer. The short cDNA fragments were subjected to end repair, adapter ligation, and agarose gel electrophoresis filtration. Then, the suitable fragments were selected as templates for PCR amplification. 6 treatments, J1Cd0, J1Cd2, J1Cd5, J77Cd0, J77Cd2 and J77Cd5, every treatment had three biological replicates. In total, eighteen cDNA libraries were constructed and sequenced using the IlluminaHiSeq^™^ 2000 platform.

### RNA-Seq data filtering, de novo assembly, gene functional annotation and classification

Clean reads were obtained by removing the adaptor sequences, duplicated sequences, ambiguous reads (N), and low-quality reads. Meantime, Q30 (sequencing error rates lower than 0.1%), GC-content and sequence duplication level of the clean data were calculated. All the downstream analyses were based on clean data with high quality. Transcriptomes of the eighteen datasets were separately assembled *de novo* using Trinity (http://trinityrnaseq.sourceforge.net/). Briefly, clean reads with a certain overlap length were initially combined to form long fragments without N that are called contigs. Related contigs were clustered using the TGICL software [21] to yield unigenes that cannot be extended on either end, and redundancies were removed to acquire non-redundant unigenes.

The assembled unigenes were searched using BLAST against the NCBI non-redundant protein sequences (NR), a manually annotated and reviewed protein sequence database (Swiss-Prot), Gene Ontology (GO), the database of Clusters of Orthologous Groups of proteins (COG), the database of Clusters of Protein homology (KOG), a database of orthologous groups of genes (eggNOG) and Encyclopedia of Genes and Genomes (KEGG) database (E ≤ 1E -5). The amino acid sequence of unigenes were predicted, and then aligned to Protein family (Pfam) database using HMMER software (http://hmmer.org/). The program was performed with an *E*-value ≤ 1E -10.

### Identification and analysis of differentially expressed genes (DEGs)

Gene expression levels were estimated by fragments per kilobase of transcript per million fragments mapped (FPKM) with Cufflink software [22]. First, the read counts for each sequenced library were adjusted by edgeR program package through one scaling normalized factor. Then, the mean of read-count of the gene from three replicate libraries was calculated as the readcount value of the gene to analyze the differences among six groups: J1Cd0 (J1Cd0-1, J1Cd0-2 and J1Cd0-3), J1Cd2 (J1Cd2-1, J1Cd2-2 and J1Cd2-3), J1Cd5 (J1Cd5-1, J1Cd5-2 and J1Cd5-3), J77Cd0 (J77Cd0-1, J77Cd0-2 and J77Cd0-3), J77Cd2 (J77Cd2-1, J77Cd2-2 and J77Cd2-3), and J77Cd5 (J77Cd5-1, J77Cd5-2 and J77Cd5-3). The DESeq R package (1.10.1) was used to identify differentially expressed genes (DEGs) between two groups according to the method described by Anders and Huber [23]. DESeq provide statistical routines for determining differential expression in digital gene expression data using a model based on the negative binomial distribution. The resulting *P*-values were adjusted using the Benjamini and Hochberg’s approach for controlling the false discovery rate (FDR). A unigene identified by DESeq with an adjusted *P*-value less than 0.05 was considered differentially expressed. DEGs were further annotated by KEGG pathway analysis. KEGG enrichment analysis was carried out in KOBAS software with a corrected *P*-value threshold of 0.05 [24].

### qRT-PCR analysis

Expression levels of 21 DEGs identified in J77Cd2 vs J77Cd0, J77Cd5 vs J77Cd0, J1Cd2 vs J1Cd0 and/or J1Cd5 vs J1Cd0 by RNA-Seq were validated using qRT-PCR analysis. Total RNA was isolated as described above. First strand cDNA fragments were synthesized using the TransScript One-Step gDNA Removal Kit (Transgen Biotech, Beijing, China). The Forward and Reverse primers designed by Primer version 5.0 (Premier Biosoft International, CA, USA) were listed in S1 Table. qRT-PCR was performed on a CFX Connect TM Optics module (Bio-Rad, CA, USA) using a TransScript Tip Green qPCR SuperMix kit (Transgen Biotech, Beijing, China) in a 25 μL reaction mixture containing 1 μL of diluted cDNA, 0.5 μM forward and reverse primers, and 12.5 μL 2 × SYBR Green PCR Master Mix. The PCR reaction protocol was 95 °C for 30 s, 40 cycles of 95 °C for 5 s and 60 °C for 30 s. All reactions were run in three biological replicates with three technical replicates. The relative expression level of the selected DEGs was calculated based on ΔΔCt algorithm using glyceraldehyde-3-phosphate dehydrogenase (c31752.graph_c1) gene as the internal standard [25]. The gene expression level in Cd-free mycelia samples was set to 1.

### Statistical analysis

Results represented as mean ± SE (n = 3). Significant differences among the six treatment combinations were analyzed by two strains × three Cd concentrations ANOVA, and followed by the Duncan’s new multiple range test.

## Results

### Effects of Cd on growth rate and Cd concentration of J1 and J77 mycelia

**Fig 1:**
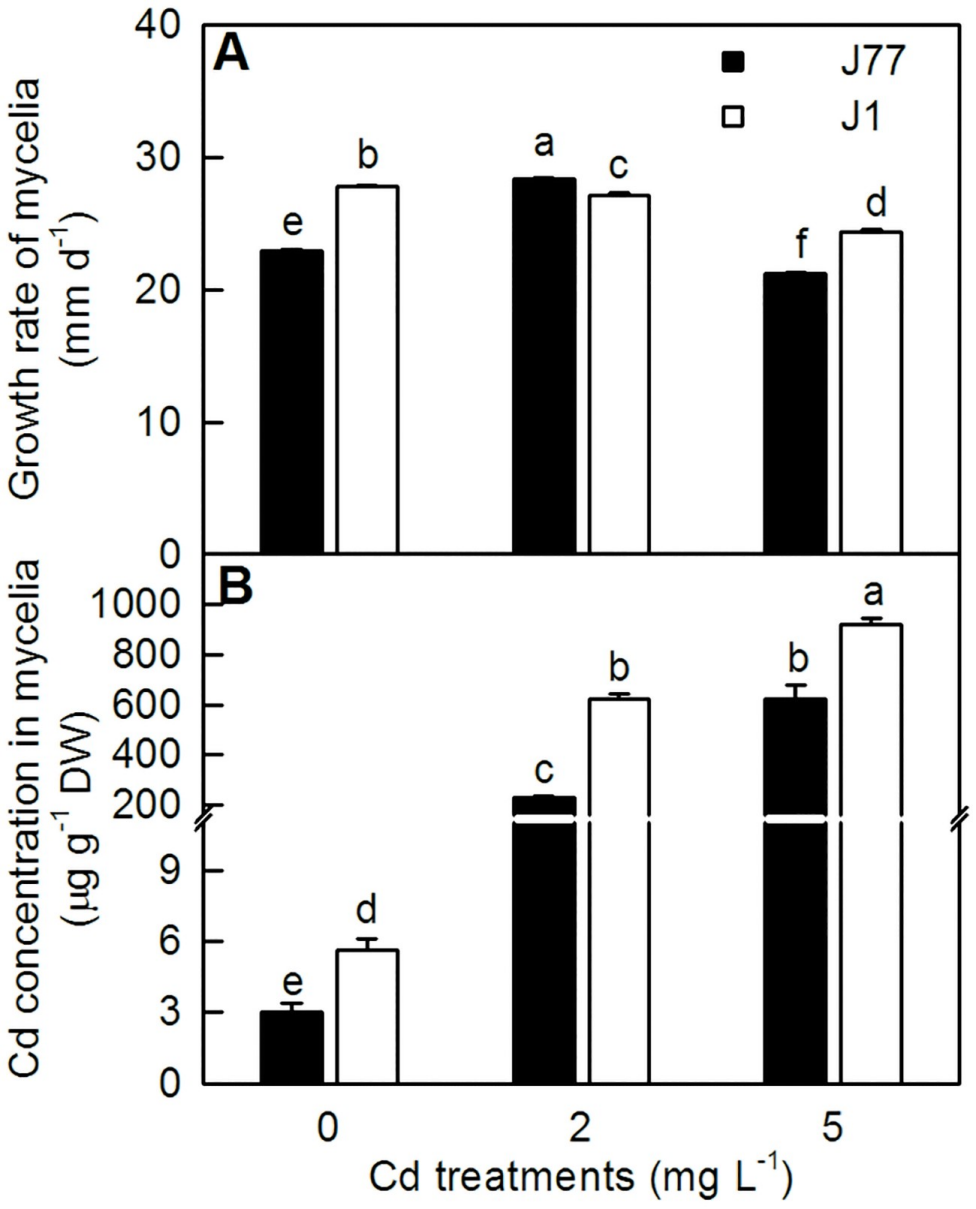
Growth rate of mycelia (a) and Cd concentration in mycelia (b) in response to Cd. Bars represent means ± SD (*n* = 3); Different letters above the bars indicate a significant difference at *P* < 0.05.

Mycelium growth rate of J1 decreased with increasing Cd concentrations. For J77, mycelium growth rate increased as Cd concentration increased at 2 mg L^-1^, and then decreased at 5 mg L^-1^(Fig 1a). Cd concentration increased with increasing Cd supply. Cd concentration was higher in J1 mycelia than that in J77 mycelia at each given Cd supply (Fig 1b).

### Illumina sequencing and transcriptome assembly

Eighteen libraries from different Cd concentration treated mycelia samples of J1 and J77 were constructed and sequenced. A total of 21027077 to 26856583 raw reads were generated from these libraries. The reads with low quality and adapters were removed, and the percentages of clean reads in all eighteen transcriptomes were above 99.18%. Q30 was more than 89% (S2 Table). Using the Trinity program, a total of 281843 putative transcripts were obtained, with an average length of 3715 bp and an N50 of 5790 bp, and transcripts with lengths of more than 500 bp accounted for 83.25% of all transcripts. The longest transcript for each locus was selected as the unigene after comparing the different transcripts representing one unigene. A total of 57884 unigenes were obtained as reference transcripts of *A*. *brasiliensis*. The mean length was 734 bp, and unigenes with lengths of more than 500 bp accounted for 28.94% of all unigenes (S3 Table).

### Functional annotation and classification of non-redundant unigenes

A total of 25091 unigenes were annotated representing 43.35% of the assembled unigenes. The remaining unigenes (56.65%) cannot be annotated with known genes, which might be caused by the presence of short sequences (41.85% < 300). In Nr, Pfam, GO, KOG and KEGG databases, 24323, 15598, 11187, 14522 and 6904 unigenes were aligned, respectively (S4 Table).

GO assignments were used to classify the functions of all predicted unigenes based on the annotations from Nr and Pfam databases. A total of 11187 unigene sequences (44.59%) were categorized into 63 functional groups consisting of 22 biological process, 20 cellular components and 21 molecular function subcategories (S1 Fig).

The sequence similarity search was performed against KOG databases to obtain the functional annotations of assembled unigenes. A total of 16517 unigene sequences (65.83%) with significant homology were assigned to 25 KOG categories (S2 Fig). The five largest groups were general function prediction only (14.39%), signal transduction mechanisms (13.65%), post-translational modification, protein turnover, chaperones (9.73%), intracellular trafficking, secretion, and vesicular transport (5.41%) and translation, ribosomal structure and biogenesis (5.12%).

### Identification and functional annotation of DEGs

**Fig 2:**
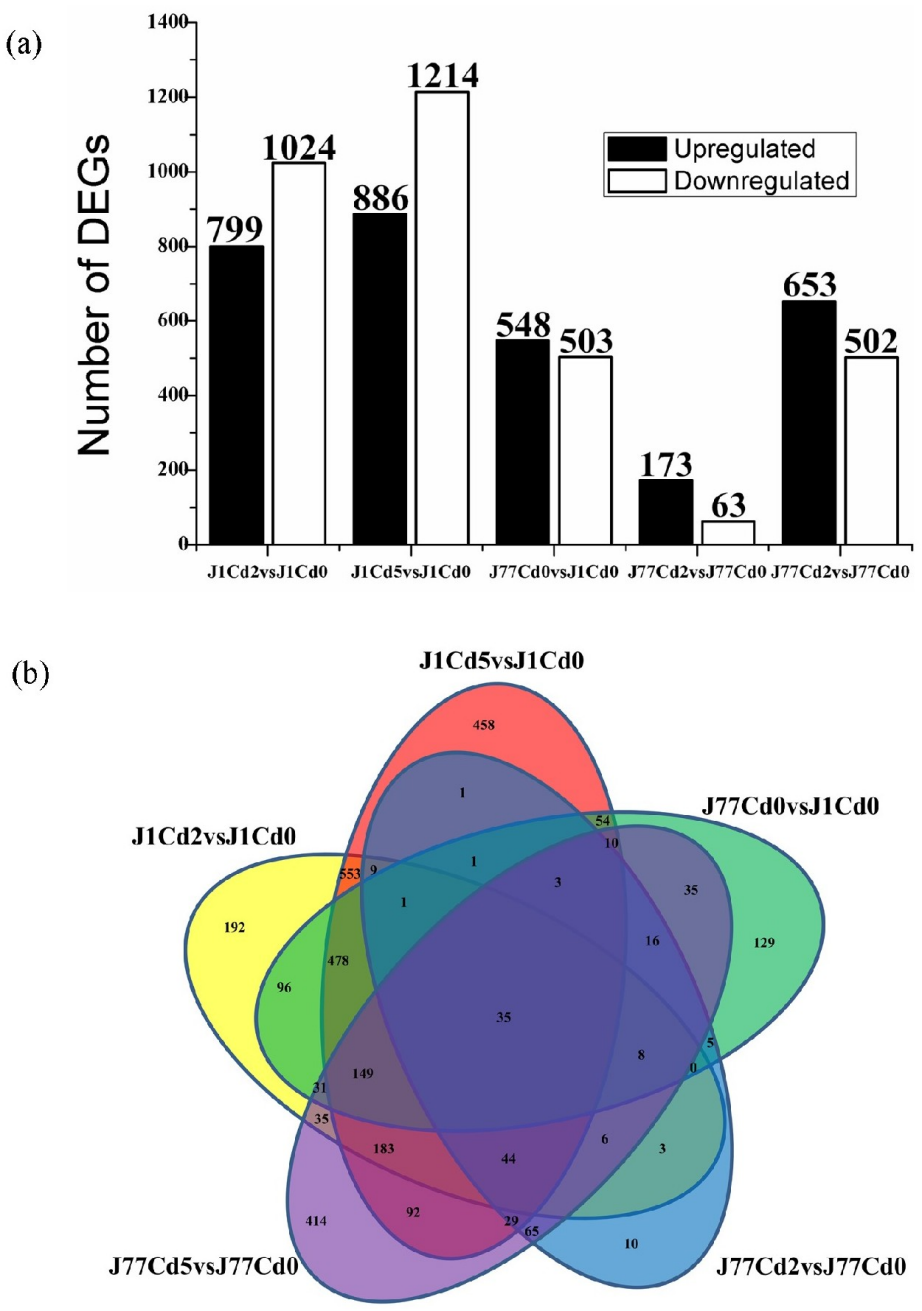
DEGs identified in Cd-treated mycelia of two *A*. *brasiliensis* strains (J1 and J77, a), and venn diagram analysis of Cd-responsive genes in J1 and J77 mycelia (b).

A gene was regarded as differentially expressed when it had an absolute value of log2 ratio ≥ 1 and a FDR ≤ 0.05. As shown in Fig 2a, we identified 799 upregulated and 1024 downregulated, 886 upregulated and 1214 downregulated, 548 upregulated and 503 downregulated, 173 upregulated and 63 downregulated, and 653 upregulated and 502 downregulated genes in J1Cd2 vs J1Cd0, J1Cd5 vs J1Cd0, J77Cd0 vs J1Cd0, J77Cd2 vs J77Cd0, and J77Cd5 vs J77Cd0, respectively. Obviously, the alterations of gene expression profiles in J1 and J77 mycelia were greater at Cd5 than those at Cd2, and both Cd2 and Cd5 affected gene expression profiles more in J1 mycelia than those in J77 mycelia. In addition, more downregulated genes than upregulated genes were identified in Cd-treated J1 mycelia, but the reverse was the case for Cd-treated J77 mycelia. As shown in Fig 2b and S5 Table, 192, 498, 129, 10 and 414 DEGs were presented only in J1Cd2 vs J1Cd0, J1Cd5 vs J1Cd0, J77Cd0 vs J1Cd0, J77Cd2 vs J77Cd0, and J77Cd5 vs J77Cd0, respectively, only 35 DEGs were identified simultaneously in J1Cd2 vs J1Cd0, J1Cd5 vs J1Cd0, J77Cd0 vs J1Cd0, J77Cd2 vs J77Cd0, and J77Cd5 vs J77Cd0. Among the 35 DEGs, 13 DEGs displayed opposite expression trends in Cd-treated J1 and J77 mycelia. Thus, Cd-induced alterations of transcriptome differ greatly between J1 and J77 mycelia.

To further characterize gene functions in terms of biological system networks, the assembled unigenes were mapped against the KEGG database, the significantly enriched KEGG pathways were present in S6 Table. As DEGs detected in J1Cd2 vs J1Cd0, no KEGG pathway was significantly enriched at a corrected *P*-value < 0.05. DEGs identified in J1Cd5 vs J1Cd0, sulfur (S) metabolism (ko00920) was significantly enriched. For DEGs detected in J77Cd0 vs J1Cd0, steroid biosynthesis (ko00100) and β-alanine metabolism (ko00410) were significantly enriched. For DEGs identified in J77Cd2 vs J77Cd0, ribosome (ko03010), β-alanine metabolism (ko00410) and galactose metabolism (ko00052) were significantly enriched. For DEGs identified in J77Cd5 vs J77Cd0, β-alanine metabolism (ko00410), histidine metabolism (ko00340), glycerolipid metabolism (ko00561), ascorbate (ASC) and aldarate metabolism (ko00053), and pentose and glucuronate interconversions (ko00040) were significantly enriched. We identified more DEGs and significantly enriched KEGG pathway in Cd5-treated J1 and J77 mycelia than those in Cd2-treated ones. Although more DEGs in Cd2- and Cd5-treated J1 mycelia than those in Cd2- and Cd5-treated J77 mycelia were identified, while more significantly enriched KEGG pathways were obtained in the latter.

### qRT-qPCR validation

To confirm the RNA-Seq expression data, we performed qRT-PCR validation for 21 DEGs selected from J17 and J77 strains. The expression profiles of all 28 data obtained from qRT-PCR are highly correlated with those from RNA-Seq (S3 Fig), demonstrating that the RNA-Seq data were reliable.

## Discussion

### J77 mycelia displayed less Cd-accumulation and higher Cd-tolerance than J1 mycelia under Cd-stress

We observed that the growth rate of J1 mycelia decreased as Cd concentration in culture media increased from 0 to 5 mg L^-1^, but the growth rate of J77 mycelia was not lower at 2 mg L^-1^ Cd than that at the absence of Cd (Fig 1a). Under Cd stress in 2 mg L^-1^, J1 and J77 showed opposite physiological phenotypes, which indicate that the two cultivars differed in the molecular mechanisms of Cd response. However, Cd in 5 mg L^-1^ has similar effect on growth rate of J1 and J77. In present study, in order to get more valuable gene information for understanding the molecular mechanism of Cd accumulation and resistance, J1 and J77 mycelia was treated by Cd in 0, 2 and 5 mg L^-1^. In addition, far less DEGs were identified in Cd-treated J77 mycelia than those in Cd-treated J1 mycelia (Fig 2a). Thus, J77 mycelia were more tolerant to Cd-stress than J1 mycelia. This might be related to the finding that Cd concentration was higher in J1 mycelia than that in J77 mycelia at each given Cd supply (Fig 1b). To conclude, J77 mycelia displayed less Cd-accumulation and higher Cd-tolerance than J1 mycelia.

### DEGs related to cellular transport

Intracellular responses of fungi to Cd include influx systems, efflux systems, and chelation of Cd by reduced glutathione (GSH), metallothioneins (MTs), and phytochelatins (PCs), followed by the transporter-mediated export or intracellular compartmentalization of the resulting complexes [26]. Toxic metals enter cells either by diffusion or by transporters which may play a role in mediating Cd influx into fungal cells across plasma membrane [27]. ZIP (Zrt, Irt-like protein-type) family which can transport divalent metal cations (such as Zn^2+^, Cd^2+^, Fe^2+^, and Cu^2+^) has been discovered in many plants, animals, fungi, protists and bacteria [28, 29]. Defect of ZIP family Zn transporter ZRT1 promoted Cd-tolerance through reducing Cd influx and alleviating Cd-induced accumulation of reactive oxygen species (ROS) and lipid in yeast (*Saccharomyces cerevisiae*) cells [30]. Here, we isolated one upregulated *ZIP Zn transporter* (c31048.graph_c0) in Cd2- and Cd5-treated J1 mycelia, but not in Cd-treated J77 mycelia (Table 1). Increased expression of *ZIP Zn transporter* gene might promote Cd uptake in Cd-treated J1 mycelia, hence enhancing Cd-accumulation in these mycelia.

**Table 1:** List of DEGs possibly involved in Cd-accumulation and Cd-tolerance of J1 and J77 mycelia.

Unigene ID	Gene annotation	Gene name	Log_2_ of fold change				
			J1Cd2 vs J1Cd0	J1Cd5 vs J1Cd0	J77Cd0 vs J1Cd0	J77Cd2 vs J77Cd0	J77Cd5 vs J77Cd0
***ABC transporters***							
c26465.graph_c0	Peroxisomal long-chain fatty acid import protein 1	PXA2	1.18	1.04	1.11		
c32662.graph_c1	Peroxisomal long-chain fatty acid import protein 2		1.05		1.00		
c28808.graph_c0	Protein SNQ2	SNQ2	-1.07	-1.43			-1.70
c30857.graph_c0	ABC transporter [Iron-sulfur clusters transporter ATM1, mitochondrial (Precursor)]	ATM1	1.31	1.08			1.25
c32453.graph_c0	Metal resistance protein YCF1	YCF1		1.57			
c31582.graph_c0	Brefeldin A resistance protein	bfr1	2.80	3.85	2.30		
c32822.graph_c0	Leptomycin B resistance protein pmd1	pmd1	1.52	1.89	1.32	-1.08	-1.31
c29937.graph_c0	Leptomycin B resistance protein pmd1	pmd1		1.95			
c29937.graph_c1	Leptomycin B resistance protein pmd1	pmd1	1.49	2.59	1.07		
c32347.graph_c0	Alpha-factor-transporting ATPase	STE6					-1.36
***Other trasnporters***							
c31048.graph_c0	ZIP Zinc transporter (Replication factor C subunit 5)		1.26	1.12			
c31926.graph_c0	Manganese-transporting ATPase 1	SPF1	1.30	1.38			
c29916.graph_c0	Cation efflux (CE) family		1.17				
*S metabolism*							
c31815.graph_c0	Putative sulfate transporter YPR003C	YPR003C	1.32	1.34	1.05		
c27405.graph_c0	Probable sulfate permease C320.05	SPCC320.05		-1.09			-1.27
c31300.graph_c0	Sulfate adenylyltransferase (ATP-sulfurylase)			1.53			1.06
**c29045.graph_c0**	**Probable 3-mercaptopyruvate sulfurtransferase**	**tum1**	**1.57**	**1.76**			**1.09**
c29356.graph_c0	Putative thiosulfate sulfurtransferase, mitochondrial (Precursor)	SPAC4H3.07c		-1.38	-1.08		
c30323.graph_c0	Adenylyl-sulfate kinase	met14	3.42	3.28			2.22
c30577.graph_c1	Sulfide:quinone oxidoreductase, mitochondrial (Precursor)	hmt2	1.26	1.39		,	
**c31374.graph_c0**	**Cystathionine gamma-synthase**	**met-7**	**1.59**	**1.36**			**1.24**
**c31522.graph_c1**	**Cysteine synthase**	**cysB**	**1.68**	**1.37**			
c32645.graph_c0	Phosphoadenosine phosphosulfate reductase	sA	2.84	1.92	2.62		-1.14
c32857.graph_c0	Sulfite reductase [NADPH] subunit beta	sir1	1.44	1.74			
c32865.graph_c0	Sulfite reductase [NADPH] flavoprotein alpha-component			1.06			
**c30654.graph_c0**	**O-acetylhomoserine (thiol)-lyase (O-Acetylhomoserine sulfhydrylase)**	**cysD**				**2.68**	**3.43**
c25130.graph_c0	Metallothionein 2		7.10	8.06	3.46	4.74	6.97
***Cys and Met metabolism***							
**c28357.graph_c0**	**Malate dehydrogenase, mitochondrial (Precursor)**	**MDH1**	**1.12**	**1.46**			
**c30948.graph_c0**	**Malate dehydrogenase, cytoplasmic**	**MDH1**	**1.52**	**1.45**			
c28741.graph_c0	S-adenosyl-L-homocysteine hydrolase		2.21	1.46	1.69		
c28857.graph_c0	S-adenosylmethionine synthase 1	L9470.9	3.54	3.04	3.21		
c28882.graph_c0	Cystathionine gamma-lyase	FUN35	1.35	1.76	1.24		
**c31374.graph_c0**	**Cystathionine gamma-synthase**	**met-7**	**1.59**	**1.36**			**1.24**
**c31522.graph_c1**	**Cysteine synthase**	**cysB**	**1.68**	**1.37**			
c29038.graph_c0	1,2-dihydroxy-3-keto-5-methylthiopentene dioxygenase 1		1.64	2.77	1.40	1.49	2.34
**c29045.graph_c0**	**Probable 3-mercaptopyruvate sulfurtransferase**	**tum1**	**1.57**	**1.76**			**1.09**
c31273.graph_c1	Probable aspartokinase (Aspartate kinase)	SPBC19F5.04	1.63	1.85			1.55
c32730.graph_c0	Probable 5-methyltetrahydropteroyltriglutamate—homocysteine methyltransferase	met26	1.90		1.37		
**c30654.graph_c0**	**O-acetylhomoserine (thiol)-lyase**	**cysD**				**2.68**	**3.43**
**c**27716.graph_c0	Methylthioribulose-1-phosphate dehydratase						2.12
***Glutathione metabolism***							
c29401.graph_c0	Glutathione synthetase large chain	gsa1					1.15
c23894.graph_c0	Protein URE2 (Glutathione S-transferase, N-terminal domain)	URE2	1.41	1.12	1.13		-1.29
c27073.graph_c0	Glutathione S-transferase		2.73		3.87		-2.24
c29002.graph_c0	Glutathione S-transferase 2	GTT2	1.62	2.96			
c29482.graph_c0	Glutathione S-transferase 2	gst2	1.24				
c30179.graph_c0	Glutathione S-transferase 2	gst2	-1.78	-1.70	-2.70		
c31193.graph_c0	Protein URE2 (Glutathione S-transferase, N-terminal domain)	URE2	3.10	3.96	1.39		1.86
c28390.graph_c0	Glutathione S-transferase 1	gst1		-1.24			
c26391.graph_c0	Glutathione S-transferase 1	gst1			-1.88		
c32644.graph_c0	Uncharacterized protein C11D3.14c	SPAC11D3.14c	1.05	1.42			
**c27411.graph_c0**	**Isocitrate dehydrogenase [NADP], mitochondrial (Precursor)**	**icdA**		**1.01**			
c28015.graph_c0	Cys-Gly metallodipeptidase dug1	dug1	1.16		1.08		
c32783.graph_c0	Putative aminopeptidase C13A11.05 (Leucyl aminopeptidase or Leucine aminopeptidase 3)	SPAC13A11.05		2.27			
c30515.graph_c0	Ribonucleoside-diphosphate reductase small chain	rnr-2	1.27	1.19			
***Cell wall metabolism***							
c31204.graph_c0	Chitin deacetylase 1 (Precursor)	CDA1	1.84	3.09			
c31045.graph_c2	Chitin deacetylase (Precursor)			1.35	1.99	-2.65	
c14349.graph_c0	Chitin deacetylase (Precursor)		4.16	3.09	3.27		
c26747.graph_c0	Chitin deacetylase (Precursor)			1.31			
c30912.graph_c0	Chitin deacetylase (Precursor)						1.49
c31888.graph_c0	Cell wall alpha-1,3-glucan synthase mok13	mok13		1.68			
c28923.graph_c0	Endochitinase 42 (Precursor)	chit42					1.27
c30790.graph_c0	Endochitinase 4 (Fragment)	chi4		-4.80			-2.58
c31056.graph_c0	Endochitinase 37 (Precursor)	chit37					2.33
c32725.graph_c2	Chitinase 1 (Precursor)	CHI1	-1.33	-1.14			
c29852.graph_c0	Exoglucanase 3 (Precursor)	cel3		-1.25			
c13117.graph_c0	Exoglucanase (Precursor)	cel2	-1.71	-1.54			
c30900.graph_c0	Endoglucanase EG-II (Precursor)	egl2	-2.24	-2.09			1.53
c31161.graph_c0	Putative endoglucanase type F (Precursor)		-4.76	-5.16	-1.83		
c19754.graph_c0	Fruiting body protein SC3 (Precursor) (Hydrophobin SC3)	SC3		-4.65			
c24051.graph_c0	Hydrophobin-3 (Precursor)	abh3	-5.31		-6.08		
c27286.graph_c0	Fruiting body protein SC3 (Precursor) (Hydrophobin SC3)	SC3	-2.39	-1.46	-2.25		
c28235.graph_c0	Hydrophobin-3 (Precursor)	abh3			-4.14	2.12	
c28830.graph_c0	Hydrophobin-3 (Precursor)	abh3		2.10			2.22
c30841.graph_c1	Hydrophobin-3 (Precursor)	abh3	2.55	2.12	-2.94		2.05
c32284.graph_c0	Fruiting body protein SC1 (Precursor) (Hydrophobin SC1)	SC1	-2.58	-3.75			
c32889.graph_c0	Hydrophobin-3 (Precursor)	abh3	-4.78	-5.41			-5.66
c13558.graph_c0	Polyphenol oxidase 2 (Precursor) (Common central domain of tyrosinase)	PPO2			2.41	-3.87	-4.01
c31420.graph_c1	Polyphenol oxidase 3 (Precursor) (Common central domain of tyrosinase)	PPO3	-1.19	-2.08	-1.55		
c30427.graph_c0	Polyphenol oxidase 2 (Precursor) (Common central domain of tyrosinase)	PPO2	-1.50	-1.17			
c32076.graph_c0	Polyphenol oxidase 4 (Precursor) (Common central domain of tyrosinase)	PPO4		-1.38			
c25127.graph_c0	Laccase-2 (Precursor)	LCC2	-5.41	-6.73			-4.86
c28781.graph_c0	Laccase (Precursor)	LCC3-1		-1.11			
c26597.graph_c0	Laccase		-2.22	-3.37			
c28781.graph_c1	Laccase-2 (Precursor)	LCC2	-1.66	-2.41			
c31020.graph_c0	Laccase-1 (Precursor)	POX1		1.56			1.04
c28668.graph_c0	Laccase-2 (Precursor)	LCC2	-1.03				-2.06
c26967.graph_c0	Laccase2b			Downregulated			
c23593.graph_c0	Laccase (Precursor)	LAC					3.29
c28957.graph_c0	Laccase-2 (Precursor)	POX2					1.95
c31141.graph_c1	Laccase-2 (Precursor)	LCC2	-1.57	-1.84			
c19686.graph_c0	Laccase (Precursor)						2.34
**c26880.graph_c0**	**UDP-glucose 6-dehydrogenase**		**2.45**	**2.35**	**1.55**		
**c30942.graph_c0**	**UDP-glucuronate decarboxylase**		**1.59**	**2.22**			
c25143.graph_c0	Cell wall integrity transcriptional regulator CAS5	CAS5					1.14
***Starch and sucrose metabolism***							
c29696.graph_c0	Putative alpha, alpha-trehalose-phosphate synthase [UDP-forming] 100 kDa subunit	SPAC22F8.05	1.36	1.76	1.14		
**c30966.graph_c0**	**Probable UTP—glucose-1-phosphate uridylyltransferase**	**fuy1**	**1.14**	**1.72**		**1.56**	**1.66**
c31314.graph_c0	Trehalose-phosphatase	tpp1		1.09			
c30621.graph_c0	Neutral trehalase	YD8119.07C	-1.57	-1.48			-1.04
c32267.graph_c0	1,4-alpha-glucan-branching enzyme	GLC3	1.08	1.03			
**c30942.graph_c0**	**UDP-glucuronate decarboxylase**		**1.59**	**2.22**			
**c26880.graph_c0**	**UDP-glucose 6-dehydrogenase**		**2.45**	**2.35**	**1.55**		
c31912.graph_c0	Amylo-alpha-1,6-glucosidase	GDB1	1.01	1.16			1.29
**c23648.graph_c0**	**Glucose-6-phosphate isomerase**	**gpi1**					**1.28**
c29537.graph_c0	Alpha-amylase 1 (Precursor)	LKA1	-3.07	-4.83	-2.06		
c30000.graph_c0	Glucoamylase (Precursor)	glaA	-2.11	-1.90	-1.24	3.35	2.97
c30613.graph_c0	Probable beta-glucosidase H	bglH	-1.48	-1.85			
c32133.graph_c0	Probable beta-glucosidase L (Precursor)	bglL	-2.04	-2.39		-1.14	-1.33
c31940.graph_c0	Probable beta-glucosidase L (Precursor)	bglL		1.21			
**c31241.graph_c0**	**Probable alpha/beta-glucosidase agdC (Precursor)**	**agdC**	**-2.07**	**-1.76**	**-1.05**	**2.21**	**3.33**
c32036.graph_c0	Probable exo-1,4-beta-xylosidase bxlB (Precursor)	bxlB		-1.31			
c31777.graph_c0	Beta-glucosidase 1B		1.02				
c28258.graph_c0	Beta-glucosidase 1B						1.53
***Pentose phosphate pathway (PPP)***							
**c19366.graph_c0**	**Fructose-bisphosphate aldolase**	**FBA1**	**1.33**	**1.02**			
c32075.graph_c1	Transaldolase	tal1		1.03			
**c32050.graph_c1**	**ATP-dependent 6-phosphofructokinase subunit beta**	**PFK2**			**1.64**	**-1.20**	**-1.19**
**c23648.graph_c0**	**Glucose-6-phosphate isomerase**	**gpi1**					**1.28**
c27170.graph_c0	Probable gluconokinase	SPAC4G9.12					1.12
c31657.graph_c1	Transketolase 1	TKL1					1.28
***Glycolysis/gluconeogenesis***							
**c23648.graph_c0**	**Glucose-6-phosphate isomerase**	**gpi1**					**1.28**
**c19366.graph_c0**	**Fructose-bisphosphate aldolase**	**FBA1**	**1.33**	**1.02**			
**c27792.graph_c0**	**Phosphoenolpyruvate carboxykinase [ATP]**	**acuF**	**1.58**	**2.21**			
c28283.graph_c0	Glyceraldehyde-3-phosphate dehydrogenase 2	gpd2	1.90	2.14			
c28694.graph_c1	Putative pyruvate decarboxylase C3G9.11c	SPAC3G9.11c	1.40	3.72			1.99
**c23819.graph_c0**	**Dihydrolipoyl dehydrogenase, mitochondrial (Precursor)**	**dld1**	**2.25**	**1.90**	**1.90**		
**c27007.graph_c0**	**Pyruvate dehydrogenase E1 component subunit alpha, mitochondrial (Precursor)**	**pda1**	**3.13**	**2.53**	**2.78**		
c31160.graph_c0	2,3-bisphosphoglycerate-independent phosphoglycerate mutase			-1.94	-2.00		
**c32902.graph_c0**	**Aldose 1-epimerase**	**gal10**		**3.37**		**5.56**	**7.55**
**c19606.graph_c0**	**Putative aldehyde dehydrogenase-like protein C922.07c**	**SPAC922.07c**		**3.15**	**2.77**		**3.48**
**c19606.graph_c1**	**Aldehyde dehydrogenase**	**aldA**					**3.92**
**c19606.graph_c2**	**Aldehyde dehydrogenase**	**aldA**	**1.85**	**3.29**	**2.58**	**2.55**	**3.67**
**c19606.graph_c3**	**Aldehyde dehydrogenase**	**aldA**		**2.99**	**2.32**	**2.56**	**3.67**
**c32143.graph_c1**	**Aldehyde dehydrogenase family**					**-1.20**	
**c29558.graph_c0**	**Aldehyde dehydrogenase**	**aldA**				**2.33**	**3.35**
**c26805.graph_c0**	**Aldehyde dehydrogenase**	**aldA**	**-3.19**	**-3.76**	**-1.26**		**-2.80**
**c28325.graph_c0**	**Aldehyde dehydrogenase**	**aldA**			**1.39**		**1.41**
**c29960.graph_c0**	Alcohol dehydrogenase 1	adh-1	-1.19	-2.33		2.36	3.46
**c32050.graph_c1**	**ATP-dependent 6-phosphofructokinase subunit beta**	**PFK2**			**1.64**	**-1.20**	**-1.19**
***Citrate cycle (TCA cycle)***							
**c27802.graph_c0**	**Citrate synthase, peroxisomal**	**CIT2**	**1.05**				
**c30708.graph_c0**	**Citrate synthase, mitochondrial (Precursor)**	**cit-1**		**1.23**			
c23960.graph_c0	Probable ATP-citrate synthase subunit 1		1.43	2.12	1.04		
c25011.graph_c0	Isocitrate dehydrogenase [NAD] subunit 2, mitochondrial (Precursor)	idh2	1.71	1.48	1.05		
**c27411.graph_c0**	**Isocitrate dehydrogenase [NADP], mitochondrial (Precursor)**	**icdA**		**1.01**			
**c28357.graph_c0**	**Malate dehydrogenase, mitochondrial (Precursor)**	**MDH1**	**1.12**	**1.46**			
**c30948.graph_c0**	**Malate dehydrogenase, cytoplasmic**	**MDH1**	**1.52**	**1.45**			
**c27792.graph_c0**	**Phosphoenolpyruvate carboxykinase [ATP]**	**acuF**	**1.58**	**2.21**			
**c27007.graph_c0**	**Pyruvate dehydrogenase E1 component subunit alpha, mitochondrial (Precursor)**	**pda1**	**3.13**	**2.53**	**2.78**		
c32195.graph_c0	2-oxoglutarate dehydrogenase, mitochondrial (Precursor)	kgd1	1.23	1.51	1.31		
c28864.graph_c0	Dihydrolipoyllysine-residue succinyltransferase component of 2-oxoglutarate dehydrogenase complex, mitochondrial (Precursor)	KGD2	2.25	1.89	1.87		
**c23819.graph_c0**	**Dihydrolipoyl dehydrogenase, mitochondrial (Precursor)**	**dld1**	**2.25**	**1.90**	**1.90**		
c32665.graph_c0	Glutamine synthetase	glnA	1.34	2.01			
***Glyoxylate and dicarboxylate metabolism***							
**c27802.graph_c0**	**Citrate synthase, peroxisomal**	**CIT2**	**1.05**				
**c30708.graph_c0**	**Citrate synthase, mitochondrial (Precursor)**	**cit-1**		**1.23**			
**c30948.graph_c0**	**Malate dehydrogenase, cytoplasmic**	**MDH1**	**1.52**	**1.45**			
**c28357.graph_c0**	**Malate dehydrogenase, mitochondrial (Precursor)**	**MDH1**	**1.12**	**1.46**			
**c28593.graph_c0**	**Malate synthase, glyoxysomal**	**acuE**	**-2.82**	**-2.13**			
c30125.graph_c0	Putative formamidase C869.04	SPAC869.04	-3.52	-3.81	-1.83		
c31536.graph_c0	Gutamine synthetase		1.14				
c32448.graph_c0	Probable serine hydroxymethyltransferase, cytosolic	SPAC24C9.12c	1.75	1.40			
c32566.graph_c0	Alanine—glyoxylate aminotransferase 1	AGX1	1.41	2.02	1.45		
**c32789.graph_c1**	**Acetyl-CoA acetyltransferase**	**PAT1**	**1.83**	**1.28**	**1.34**		
c30820.graph_c0	Protein rer1	rer1		1.07			
***Pyruvate metabolism***							
**c30948.graph_c0**	**Malate dehydrogenase, cytoplasmic**	**MDH1**	**1.52**	**1.45**			
**c28357.graph_c0**	**Malate dehydrogenase, mitochondrial (Precursor)**	**MDH1**	**1.12**	**1.46**			
**c28593.graph_c0**	**Malate synthase, glyoxysomal**	**acuE**	**-2.82**	**-2.13**			
**c**29626.graph_c0	NAD-dependent malic enzyme, mitochondrial (Precursor)	MAE1	1.13				
c29626.graph_c1	NAD-dependent malic enzyme, mitochondrial (Precursor)	MAE1	2.20	2.06	1.43		1.08
**c27792.graph_c0**	**Phosphoenolpyruvate carboxykinase [ATP]**	**acuF**	**1.58**	**2.21**			
**c27007.graph_c0**	**Pyruvate dehydrogenase E1 component subunit alpha, mitochondrial (Precursor)**	**pda1**	**3.13**	**2.53**	**2.78**		
**c23819.graph_c0**	**Dihydrolipoyl dehydrogenase, mitochondrial (Precursor)**	**dld1**	**2.25**	**1.90**	**1.90**		
**c26896.graph_c0**	Cytochrome b2, mitochondrial (Precursor)	CYB2	-1.27	-1.04			
**c32789.graph_c1**	**Acetyl-CoA acetyltransferase**	**PAT1**	**1.83**	**1.28**	**1.34**		
**c19606.graph_c0**	**Putative aldehyde dehydrogenase-like protein C922.07c**	**SPAC922.07c**		**3.15**	**2.77**		**3.48**
**c19606.graph_c1**	**Aldehyde dehydrogenase**	**aldA**					**3.92**
**c19606.graph_c2**	**Aldehyde dehydrogenase**	**aldA**	**1.85**	**3.29**	**2.58**	**2.55**	**3.67**
**c19606.graph_c3**	**Aldehyde dehydrogenase**	**aldA**		**2.99**	**2.32**	**2.56**	**3.67**
**c29558.graph_c0**	**Aldehyde dehydrogenase**	**aldA**				**2.33**	**3.35**
**c32143.graph_c1**	**Aldehyde dehydrogenase family**					**-1.20**	
**c26805.graph_c0**	**Aldehyde dehydrogenase**	**aldA**	**-3.19**	**-3.76**	**-1.26**		**-2.80**
**c28325.graph_c0**	**Aldehyde dehydrogenase**	**aldA**			**1.39**		**1.41**
***Fructose and mannose metabolism***							
**c19366.graph_c0**	**Fructose-bisphosphate aldolase**	**FBA1**	**1.33**	**1.02**			
c30725.graph_c0	Fructose-2,6-bisphosphatase	FBP26	1.37		1.14		
c26689.graph_c0	Phosphomannomutase	PMM1	1.55	1.52		1.17	1.05
c30639.graph_c1	L-galactonate dehydratase	lgd1		1.11			
**c32050.graph_c1**	**ATP-dependent 6-phosphofructokinase subunit beta**	**PFK2**			**1.64**	**-1.20**	**-1.19**
***Galactose metabolism***							
**c30966.graph_c0**	**Probable UTP—glucose-1-phosphate uridylyltransferase**	**fuy1**	**1.14**	**1.72**		**1.56**	**1.66**
c31270.graph_c0	Probable alpha-galactosidase B (Precursor)		1.50		1.45		-1.10
**c32902.graph_c0**	**Aldose 1-epimerase**	**gal10**		**3.37**		**5.56**	**7.55**
**c32050.graph_c1**	**ATP-dependent 6-phosphofructokinase subunit beta**	**PFK2**			**1.64**	**-1.20**	**-1.19**
**c31241.graph_c0**	**Probable alpha/beta-glucosidase agdC (Precursor)**	**agdC**	**-2.07**	**-1.76**	**-1.05**	**2.21**	**3.33**
c30922.graph_c0	Galactose-1-phosphate uridylyltransferase	GAL7		-1.09			
c26976.graph_c0	Probable alpha-galactosidase B (Precursor)	aglB				-1.50	-1.80
c29404.graph_c0	L-galactonate dehydratase	lgd1					1.29
***Pentose and glucuronate interconversions***							
**c19606.graph_c0**	**Putative aldehyde dehydrogenase-like protein C922.07c**	**SPAC922.07c**		**3.15**	**2.77**		**3.48**
**c19606.graph_c1**	**Aldehyde dehydrogenase**	**aldA**					**3.92**
**c19606.graph_c2**	**Aldehyde dehydrogenase**	**aldA**	**1.85**	**3.29**	**2.58**	**2.55**	**3.67**
**c19606.graph_c3**	**Aldehyde dehydrogenase**	**aldA**		**2.99**	**2.32**	**2.56**	**3.67**
**c29558.graph_c0**	**Aldehyde dehydrogenase**	**aldA**				**2.33**	**3.35**
**c26805.graph_c0**	**Aldehyde dehydrogenase**	**aldA**	**-3.19**	**-3.76**	**-1.26**		**-2.80**
**c28325.graph_c0**	**Aldehyde dehydrogenase**	**aldA**			**1.39**		**1.41**
**c26880.graph_c0**	**UDP-glucose 6-dehydrogenase**		**2.45**	**2.35**	**1.55**		
**c30966.graph_c0**	**Probable UTP—glucose-1-phosphate uridylyltransferase**	**fuy1**	**1.14**	**1.72**		**1.56**	**1.66**
***Cell cycle-yeast***							
c31614.graph_c0	Anaphase-promoting complex subunit 2	apc2	-1.00				
c29009.graph_c0	DASH complex subunit DAM1	DAM1		-1.08			
c27669.graph_c0	Mitotic spindle checkpoint component mad2	mad2		-1.22			
c27963.graph_c0	F-box and WD-40 domain protein CDC4 (Cell division control protein 4)	CDC4	-1.42				
c29933.graph_c0	F-box and WD-40 domain protein CDC4 (Cell division control protein 4)	CDC4		1.67			
c31292.graph_c1	WD repeat-containing protein slp1	slp1	1.83	2.75	1.18		1.14
c30413.graph_c0	Condensin complex subunit 1	cnd1	1.75	1.75	1.37		
c27985.graph_c0	Condensin complex subunit 2	cnd2	1.38	1.57			1.05
c31854.graph_c0	Condensin complex subunit 3	cnd3	1.30	1.67	1.02		
c29241.graph_c0	Transcriptional repressor rco-1	rco-1	1.38	2.28			
c31456.graph_c0	Separin	cut1		1.62			
c32406.graph_c0	Protein TSD2	TSD2		1.21			
c31184.graph_c0	Serine/threonine-protein kinase mph1	mph1	2.59	1.64	3.14		-1.32
***Mismatch repair***							
**c29386.graph_c0**	**Replication factor A1**			**-1.97**	**1.68**		**1.39**
**c29976.graph_c0**	**Replication factor C subunit 2**	**RFC2**	**-1.39**	**-1.52**	**-1.89**		
**c28292.graph_c0**	**Replication factor A protein 1**	**ssb1**	**1.00**	**1.00**			
**c24853.graph_c0**	**Replication factor A1**						**3.71**
**c29680.graph_c0**	**Replication factor C subunit 3**	**rfc3**					**1.69**
**c30247.graph_c0**	**ATP dependent DNA ligase domain**		**1.69**	**2.12**	**1.37**		**1.32**
**c31357.graph_c1**	**DNA-directed RNA polymerase II subunit rpb7**	**rpb7**					**1.04**
***Base excision repair***							
**c30247.graph_c0**	**ATP dependent DNA ligase domain**		**1.69**	**2.12**	**1.37**		**1.32**
**c31357.graph_c1**	**DNA-directed RNA polymerase II subunit rpb7**	**rpb7**					**1.04**
**c32763.graph_c1**	**DNA polymerase epsilon catalytic subunit A**	**POL2**	**1.67**	**2.43**	**1.79**		
**c28697.graph_c0**	**XPG N-terminal domain**		**1.50**	**1.43**	**1.27**		
c31675.graph_c0	Flap endonuclease 1-A						1.26
c29597.graph_c0	A/G-specific adenine DNA glycosylase	myh1	1.49	1.28	1.58		
c19089.graph_c0	Uracil-DNA glycosylase						4.05
c28239.graph_c0	Endonuclease III homolog						1.24
c30805.graph_c1	DNA-(apurinic or apyrimidinic site) lyase 2	apn2					-1.12
***Nucleotide excision repair***							
**c29386.graph_c0**	**Replication factor A1**			**-1.97**	**1.68**		**1.39**
**c29976.graph_c0**	**Replication factor C subunit 2**	**RFC2**	**-1.39**	**-1.52**	**-1.89**		
**c28292.graph_c0**	**Replication factor A protein 1**	**ssb1**	**1.00**	**1.00**			
**c24853.graph_c0**	**Replication factor A1**						**3.71**
**c29680.graph_c0**	**Replication factor C subunit 3**	**rfc3**					**1.69**
**c30247.graph_c0**	**ATP dependent DNA ligase domain**		**1.69**	**2.12**	**1.37**		**1.32**
**c31357.graph_c1**	**DNA-directed RNA polymerase II subunit rpb7**	**rpb7**					**1.04**
**c32763.graph_c1**	**DNA polymerase epsilon catalytic subunit A**	**POL2**	**1.67**	**2.43**	**1.79**		
c32218.graph_c0	DNA repair protein rhp42	rhp42	1.12		1.57		
c27368.graph_c0	DNA repair helicase rad15	rad15		1.36		1.46	1.24
***DNA replication***							
**c29386.graph_c0**	**Replication factor A1**			**-1.97**	**1.68**		**1.39**
**c29976.graph_c0**	**Replication factor C subunit 2**	**RFC2**	**-1.39**	**-1.52**	**-1.89**		
**c28292.graph_c0**	**Replication factor A protein 1**	**ssb1**	**1.00**	**1.00**			
**c24853.graph_c0**	**Replication factor A1**						**3.71**
**c29680.graph_c0**	**Replication factor C subunit 3**	**rfc3**					**1.69**
**c30247.graph_c0**	**ATP dependent DNA ligase domain**		**1.69**	**2.12**	**1.37**		**1.32**
**c31357.graph_c1**	**DNA-directed RNA polymerase II subunit rpb7**	**rpb7**					**1.04**
**c32763.graph_c1**	**DNA polymerase epsilon catalytic subunit A**	**POL2**	**1.67**	**2.43**	**1.79**		
**c28697.graph_c0**	**XPG N-terminal domain**		**1.50**	**1.43**	**1.27**		
**c31675.graph_c0**	**Flap endonuclease 1-A**						**1.26**
c13106.graph_c0	Ribonuclease H	rnh1					3.72

DEGs presented in two or more pathways were marked in bold.

DEGs in red font were key candidate genes related to cadmium accumulation and/or tolerance.

Manganese (Mn)-transporting ATPase 1 (SPF1, Sensitivity to *Pichia* *f**arinosa* killer toxin 1) gene which encodes a highly conserved, endoplasmic reticulum (ER) localized, putative P-type ATPase was induced by Cd in J1 mycelia, but not in J77 mycelia (Table 1). Cohen et al. [31] observed a reduction in the concentration of Mn^2+^ in the ER lumen of *Δspf1* yeast cells and an increase following its overexpression, indicating that SPF1 was involved in the regulation of Mn transport into ER. SPF1 is one of the two yeast P5 ATPases, along with vacuolar P-type ATPase Ypk9 (the closest homologue of SPF1). Deletion of *Ypk9p* caused sensitivity against Cd, Mn, selenium (Se) and nickel (Ni) [32]. Thus, SPF1 might also be involved in Cd transport into ER. The upregulation of *SPF1* might play a role in the Cd-tolerance of J1 mycelia by enhancing Cd sequestration in ER.

ATP-binding cassette (ABC) transporters, which catalyze the ATP-dependent transport of a broad range of compounds across biological membranes, are involved in Cd-tolerance in eukaryotic cells [33]. *ATM1* is a GSH-dependent half-size ABC transporter that exports Fe-S clusters from mitochondrial matrix into cytosol [34]. Hanikenne et al. [35] found that a mitochondrial ATM-like transporter gene in *Chlamydomonas reinhardtii* was strongly induced when cells submitted to Cd-stress, and its deficient cells were hypersensitive to Fe and Cd-stress. They suggested that the mitochondrial ATM-like transporter played a key role in the Cd-tolerance of *C*. *reinhardtii*, possibly through the export of Cd outside the mitochondrial matrix, thus protecting the mitochondrial function from Cd-toxicity and/or the modification of Fe homeostasis in the algal cells. In this study, we observed that the expression level of *ATM1* was upregulated in Cd2- and Cd5-treated J1 mycelia, but not in Cd-treated J77 mycelia (Table 1). The upregulation of *ATM1* in Cd-treated J1 mycelia might be involved in Cd-tolerance by exporting Cd outside the mitochondrial matrix and/or modifying Fe homeostasis. *ATM1* expression level was significantly higher in J77 mycelia than that in J1 mycelia at Cd0. This could explain why *ATM1* expression was not significantly upregulated in Cd-treated J77 mycelia. In yeast, *YCF1* is responsible for the transport of GSH-complexes from cytosol into vacuole. Its expression was induced by Cd [27]. Here, the expression level of *YCF1* was elevated in Cd5-treated J1 mycelia (Table 1). This could be explained as more increased requirement for the removal of cytosol Cd into vacuole, since Cd concentration was higher in J1 mycelia than that in J77 mycelia when exposed to Cd (Fig 1b). Also, we identified several differentially expressed ABC transporter genes related to secondary metabolites biosynthesis, transport and catabolism in J1Cd2 vs J1Cd0 [viz. *SNQ2*, *pmd1* (c29937.graph_c1), *bfr1* and *pmd1* (c32822.graph_c0)], J1Cd5 vs J1Cd0 [viz. *SNQ2*, *bfr1*, *pmd1* (c32822.graph_c0), *pmd1* (c29937.graph_c0) and *pmd1* (c29937.graph_c1)], J77Cd0 vs J1Cd0 [viz. *bfr1*, *pmd1* (c32822.graph_c0) and *pmd1* (c29937.graph_c1)], J77Cd2 vs J77Cd0 [viz. *pmd1* (c32822.graph_c0)] and J77Cd5 vs J77Cd0 (viz. *SNQ2*, *pmd1* (c32822.graph_c0) and *STE6*] and related to lipid transport and metabolism in J1Cd2 vs J1Cd0 (viz. *PXA2* and c32662.graph_c1), J1Cd5 vs J1Cd0 (viz. *PXA2*) and J77Cd0 vs J1Cd0 (viz. *PXA2* and c32662.graph_c1). Therefore, ABC transporters might be involved in the Cd-tolerance of *A*. *brasiliensis* mycelia.

Cation efflux (CE) family, also known as the cation diffusion facilitator (CDF) family, can either sequester metal ions within cells or export metal ions out of cells in organisms. Macdiarmid et al. [36] observed that CDF family proteins Zrc1 and Cot1 might transport Cd, Co and Zn into vacuoles of yeast. Yeast mutants deficient in *Zrc1* and *Cot1* were hypersensitive to Cd and Zn, or Co and Ni [37]. Here, we isolated one upregulated CE family (c29916.graph_c0) gene in Cd2-treated J1 mycelia (Table 1), indicating that the sequestration of Cd into vacuoles might be increased in these mycelia.

### DEGs related to S, cysteine, methionine and glutathione metabolisms

S-containing compounds biosynthesized in S metabolism, including H_2_S, cysteine (Cys), GSH, PCs, and MTs play key roles in the detoxification of Cd and other heavy metals and the alleviation of oxidative stress in organisms including fungi [38–40]. Kennedy et al. [41] found that *Schizosaccharomyces pombe* strains mutated in genes involved in S assimilation [viz. *sulfite reductase* (*SiR*), *siroheme synthase*, *3'-phosphoadenylylsulfate reductase* (*PAPS reductase*), *uncharacterized FAD-binding protein C12C2*.*03c*, *sulfide-quinone oxidoreductase* (*SQR*), *adenylylsulfate kinase* (*APS kinase*) and *ATP sulfurylase* (*ATPS)*], Cys (viz. *Cys synthase*) and PC (viz. *PC synthase*) biosynthesis, and glutathione metabolism (viz. *glutamate-Cys ligase* and *zinc metalloprotease*) were sensitive to Cd. H_2_S, as a messenger molecule, is involved in many physiological processes in organisms. For example, H_2_S can protect neurons against oxidative damage by increasing GSH production due to both enhanced activity of γ-glutamylcysteine synthetase and transport of Cys [42]. *Escherichia coli* overexpressed *AtLCD* and *AtDCD* from *Arabidopsis thaliana* involved in H_2_S biosynthesis had higher H_2_S production rate and resistance to Cd-toxicity, and less oxidative damage [43]. Sun et al. (2013) observed that H_2_S alleviated Cd toxicity via improving antioxidant system, decreasing Cd influx through the H_2_O_2_-activiated plasma membrane (PM) Ca channels, and increasing the sequestration of Cd in the vacuole presumably through the activation of tonoplast Cd^2+^/H^+^ antiporters Populus euphratica cells [44].

Cys is one of substrates for the elevated biosynthesis of Cd-sequestering compounds such as GSH, PCs and MTs in organisms [18, 27]. In fungi, Cys biosynthesis involves ATPS, APS kinase, PAPS reductase, SiR, O-acetylhomoserine sulfhydrylase, Cys synthase and cystathionine γ-lyase [45]. Also, Cys is one of the substrates for methionine (Met) biosynthesis. Cystathionine γ-synthase (CgS) catalyzes the first committed reaction of Met biosynthesis to form cystathionine from Cys. The yielding cystathionine is cleaved to produce homocysteine, which is then methylated by Met synthase (MS) to form Met. Met can serve as a precursor for protein and S-adenosylmethionine (SAM) biosynthesis. The biosynthesis of SAM from Met and ATP is catalyzed by SAM synthase (SAMS).

GSH, the most abundant nonprotein thiol component of eukaryotic cells and free radical scavenger, plays a role in the sequestration of heavy metals and detoxification of ROS and xenobiotics. GSH biosynthesis, starting from inorganic sulfate, requires both the S assimilation and the Cys biosynthetic pathways [45]. GSH biosynthesis is catalyzed by two ATP dependent enzymes γ-glutamylcysteine synthetase (GSH1) and glutathione synthetase (GSH2). In addition to GSH biosynthesis, GSH-mediated Cd sequestration also depends on a rapid formation of GSH conjugates with Cd^2+^, which can be catalyzed by glutathione S-transferases (GSTs) [46]. GSTs play important roles in protecting cells from Cd-induced oxidative stresses through scavenging reactive molecules with the addition of GSH. Many GSTs can function as glutathione peroxidases. Gomes et al. [47] reported that a yeast mutant deficient in the synthesis of GSH (*gsh2*) displayed enhanced Cd uptake, low level of intracellular oxidation, and normal growth up to 50 mg L^-1^ CdSO_4_. Adamis et al. [46] found that yeast cells mutated in GST I and II (GTT1 and GTT2) genes had twice as much Cd uptake than the control strain, but the three strains displayed normal growth at 48 μM CdSO_4_. Indeed, *Δgtt2* cells had higher tolerance to Cd than controls. Further study showed that addition of GSH monoethyl ester (GME, a cell-permeable derivative of GSH) decreased Cd uptake in control and *Δgtt1* strains, but not in *Δgtt2* strains, indicating that GTT1 and GTT2 might be involved in the regulation of GSH homeostasis and the formation of the GSH-Cd complex, respectively. Here, we isolated one unregulated glutathione synthetase large chain gene (*gsa1*) in Cd5-treated J77 mycelia, but not in Cd-treated J1 mycelia; and four upregulated genes involved in GSH biosynthesis (viz. *SPAC11D3*.*14c* and *icdA*) and degradation (viz. *dug1* and *SPAC13A11*.*05*) in Cd2- and/or Cd5-treated J1 mycelia, but not in Cd-treated J77 mycelia. Also, we identified more upregulated than downregulated GST genes in Cd-treated J1 mycelia, but more downregulated than upregulated GST genes in Cd-treated J77 mycelia (Table 1). Thus, the differences in Cd-induced alterations of GST genes and *gsa1* between J77 and J1 mycelia might be responsible for the less Cd uptake and higher Cd-tolerance of J77 mycelia.

PCs can react with Cd by GST in cytosol and then they are sequestered into vacuole for degradation. Their biosynthesis is enhanced by Cd, and deletion mutants display hypersensitivity to Cd [27]. Weghe and Ow (1999) observed that *HMT2* encoding a mitochondrial SQR responsible for sulfide oxidation played an important role in the detoxification of excess sulphide produced under Cd-stress and the biosynthesis of PCs in *S*. *pombe* [48].

MTs can protect cells against Cd-toxicity by binding Cd, then either export or compartmentalization in vacuole [26]. According to the arrangement of Cys residues, MTs can be subdivided into 3 classes: Class I MTs, which are mainly found in vertebrates, Class II MTs are mainly found in plants, fungi and invertebrates, and Class III MTs (viz. PCs) [49]. Courbot et al. [38] reported that the concentrations of GSH, γ-glutamylcysteine (the direct precursor for both GSH and PCs) and a 3-kDa molecular mass (most probably related to a MT) were increased in Cd-treated mycelia of ectomycorrhizal fungus *Paxillus involutus*. Here, *metallothionein 2* (*MT2*) was induced in Cd-treated J1 and J77 mycelia. Moreover, its expression level was higher in J77 mycelia than that in J1 mycelia at each given Cd supply (Table 1). Thus, *MT2* might play an important role in Cd sequestration of *A*. *brasiliensis*, and contribute to the difference in Cd-tolerance between the two strains.

**Fig 3:**
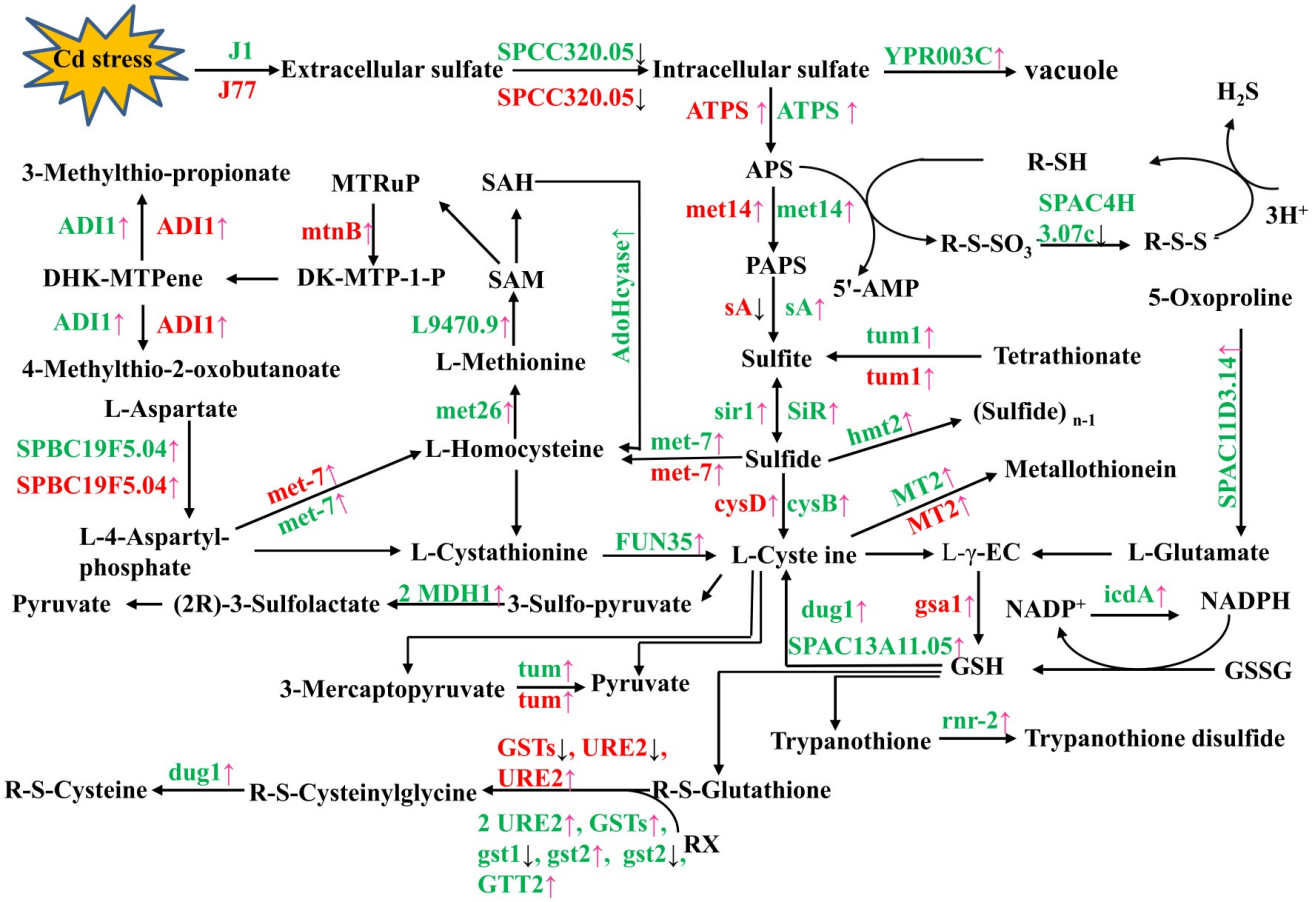
DEGs involved in sulfur metabolism (ko00920), cysteine and methionine metabolism (ko00270) and glutathione metabolism (ko00480) in Cd2- and/or Cds-treated J1 and J77 mycelia. Pink arrow: upregulation; Dark arrow: downregulation; ADI1: 1,2-dihydroxy-3-keto-5-methylthiopentene dioxygenase; AdoHcyase: S-adenosyl-L-homocysteine hydrolase; 5ꞌ-AMP: 5ꞌ-Adenosine monophosphate; APS: 5′-phosphosulfate; ATPS: sulfate adenylyltransferase; DK-MTP-1-P: 2,3-diketo-5-methylthiopentyl-1-phosphate; GSH: reduced glutathione; GSSG: oxidized glutathione; GST: glutathione S-transferase; L-γ-EC: L-γ-Glutamylcysteine; MTRuP: S-methyl-5-thio-D-ribulose-1-phosphate; PAPS: 3'-phosphoadenosine-5'-phosphosulfate; mtnB: methylthioribulose-1-phosphate dehydratase; SAM: S-adenosyl-L-methionine; SAH: S-adenosylhomocysteine.

In conclusion, S, Cys, Met and glutathione metabolisms were upregulated in Cd-regulated J1 and J77 mycelia, especially in the former (Table 1 and Fig 3). This agreed with the more increased demand for the detoxification of Cd in Cd-treated J1 mycelia, because its concentration was higher in Cd-treated J1 mycelia than that in Cd-treated J77 mycelia. It is worth noting that the expression levels of quite a few genes involved in these metabolisms were higher in J77 mycelia than those in J1 mycelia at Cd0, and their expression was not induced by Cd. This might contribute to the higher Cd-tolerance of J77 mycelia.

### DEGs related to cell wall

Fungal cell wall mainly consists of chitin, chitosan (deacetylated chitin), glucan, and various mucopolysaccharides. Fungal cell wall polysaccharide binding to heavy metals is one of the important detoxification mechanisms in fungi. Bhanoori and Venkateswerlu [50] indicated that Cd-induced increase of chitin content in cell wall might be an adaptive strategy of *Neurospora crassa* to mitigate the toxic effects of Cd-accumulation through increased chitin-Cd complexation in cell wall. Also, fungal cell wall may be actively modified when exposed to Cd. Wang et al. [14] found that *chitin deacetylase* involved in chitosan biosynthesis was induced by Cd in *A*. *brasiliensis*. Zhao et al. [19] reported that *chitinase* involved in chitin degradation was inhibited in Cd-treated fungus *Exophiala pisciphila*. In present study, we isolated four upregulated *chitin deacetylases* (viz. *CDA1*, c31045.graph_c2, c14349.graph_c0 and c26747.graph_c0) and one upregulated *mok13* involved in cell wall polysaccharide biosynthesis, and seven downregulated genes [viz. three chitinase (*chit42*, *chi4* and *chit37*) and four glucanase (*cel3*, *cel2*, *egl2* and c31161.graph_c0)] involved in cell wall polysaccharide degradation in J1Cd2 vs J1Cd0 and/or J1Cd5 vs J1Cd0, but only one downregulated (c31045.graph_c2) and one upregulated (c30912.graph_c0) chitin deacetylase gene in J77Cd2 vs J77cd0 and J77Cd5 vs J77Cd0, respectively, and one downregulated *chi4* and one upregulated *chit42* in J77Cd5 vs J77Cd0 (Table 1). This implies that cell wall polysaccharide biosynthesis and degradation was increased and decreased in Cd-treated J1 mycelia, respectively, which could enhance the levels of cell wall polysaccharides; but was far less affected in Cd-treated J77 mycelia. This agreed with the more increased requirement for Cd chelation in the cell wall of Cd-treated J1 mycelia.

Hydrophobins are small, cell-wall-associated proteins rich in Cys and with low sequence similarity. Jacob et al. [18] observed that two *hydrophobins* were downregulated and one *MT* was induced by Cd in ectomycorrhizal fungus *Paxillus involutus*, suggesting that hydrophobin biosynthesis might be decreased, thus redirecting S to the production of MTs. This could explain why more downregulated than upregulated hydrophobin genes identified in Cd-treated J1 mycelia. Interestingly, we identified six upregulated and two downregulated hydrophobin genes in J77Cd2 vs J77Cd0, but only three downregulated hydrophobin genes in J77Cd5 vs J77Cd0 (Table 1).

Melanins, which present mainly in cell wall, can bind Cd [51]. Tyrosinase is the rate-limiting enzyme for melanin biosynthesis, while many of the less specific polyphenol oxidases (PPOs) such as laccases may catalyze the formation of melanins [14, 27]. Blaudez et al. (2000) suggested that Cd-induced upregulation of *tyrosinase* in *P*. *involutus* mycelia was an adaptive mechanism by increasing Cd sequestration onto cell-wall pigments due to enhanced melanin biosynthesis [51]. Also Jacob et al. [18] observed that Cd-induced increases in laccase activity and production of malanins were involved in the Cd-tolerance of *P*. *involutus* mycelia. In this study, we isolated three downregulated PPO (common central domain of tyrosinase) genes and seven downregulated *laccases* from J1Cd2 vs J1Cd0 and/or J1Cd5 vs J1Cd0, but only one upregulated *laccase* from J1Cd5 vs J1Cd0. However, we isolated one downregulated *PPO* from J77Cd2 vs J77Cd0 and J77Cd5 vs J7Cd0, and two downregulated and four upregulated *laccases* from J77Cd5 vs J7Cd0 (Table 1). So, melanin biosynthesis might be decreased in Cd-treated J1 mycelia, but less affected in Cd-treated J77 mycelia which might be responsible for the higher Cd-tolerance of J77 mycelia.

UDP-glucose 6-dehydrogenase (UGDH) catalyzes UDP-glucose into UDP-glucuronic acid (UDP-GlcA), a constituent of complex glycosaminoglycans (acid mucopolysaccharides). UDP-GlcA decarboxylase (UXS), which catalyzes the decarboxylation of UDP-GlcA to UDP-xylose, is required for the biosynthesis of the core tetrasaccharide in glycosaminoglycan biosynthesis. Thus, the upregulation of *UGDH* and *UXS* (viz. c26880.graph_c0 and c30942.graph_c0) in Cd-treated J1 mycelia (Table 1) implied that glycosaminoglycan biosynthesis might be enhanced in these mycelia [52].

Cell wall integrity transcriptional regulator CAS5 acts with transcriptional adapter 2 to enhance cell wall integrity [53]. The upregulation of *CAS5* in Cd5-treated J77 mycelia (Table 1) indicated that J77 mycelia might have higher capacity to maintain cell wall integrity, thus enhancing the Cd-tolerance.

Fungal cell wall not only plays a key role in the defense to withstand stressful environments, but also is vital for cell division, cell growth and development [53]. Here, the expression levels of many genes involved in cell wall metabolism were altered in Cd-treated J1 mycelia, but far less were affected in Cd-treated J77 mycelia. Therefore, Cd-induced alterations of cell wall might be responsible for the inhibited J1 mycelium growth.

### DEGs related to carbohydrate metabolism

**Fig 4:**
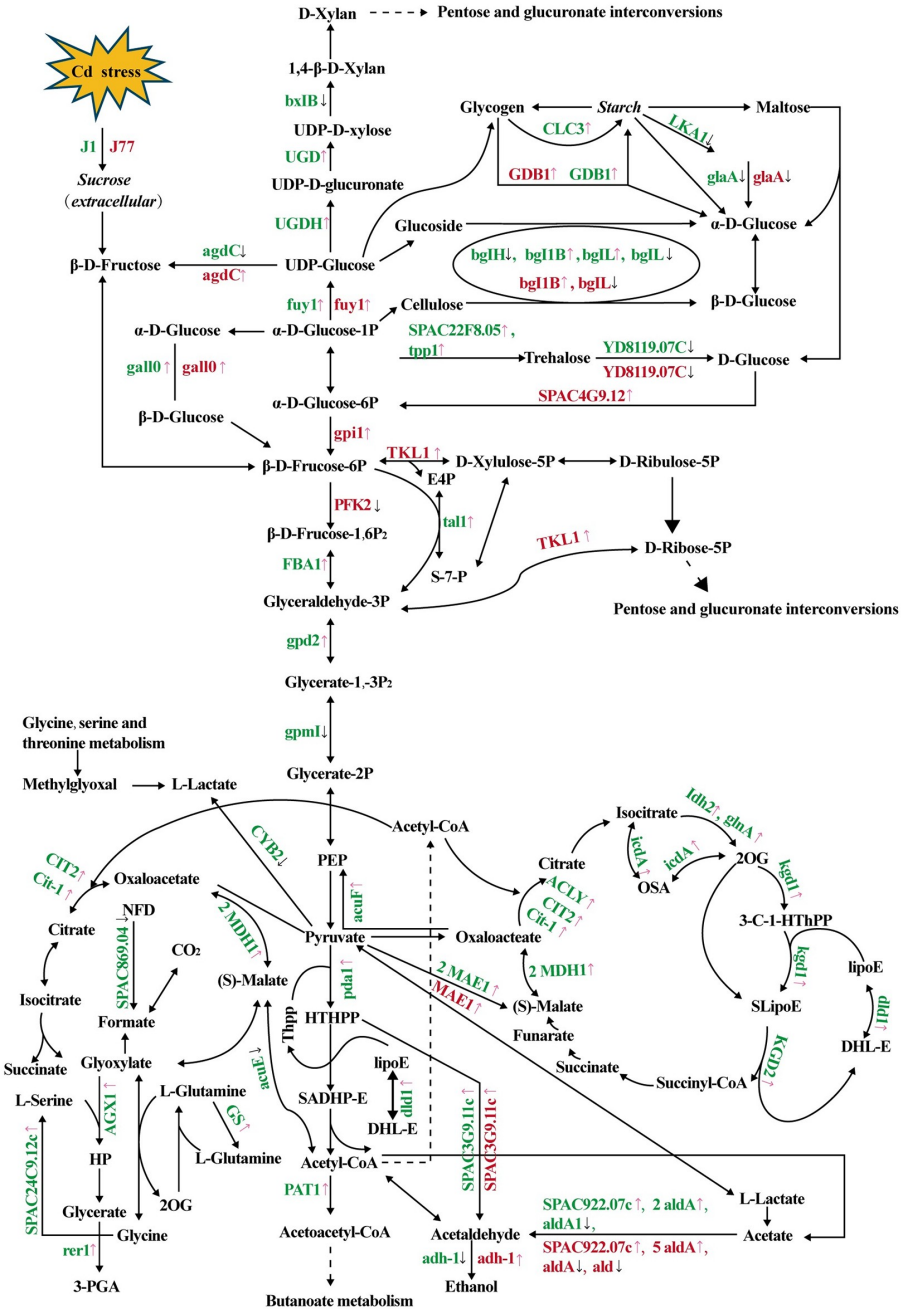
DEGs involved in starch and sucrose metabolism (ko00500), pentose phosphate pathway (ko00030), glycolysis/gluconeogenesis (ko00010), TCA cycle (ko00020), glyoxylate and dicarboxylate metabolism (ko00630), pyruvate metabolism (ko00620) in Cd2- and/or Cd5-treated J1 and J77 mycelia. Pink arrow: upregulation; Dark arrow: downregulation; ACLY: probable ATP-citrate synthase subunit 1; ald: aldehyde dehydrogenase family; bgl1B: β-glucosidase 1B; 3-C-1-HThPP: 3-carboxy-1-hydroxypropyl-Thpp; DHL-E: dihydrolipoamide-E; E4P: D-erythrose-4P; GS: gutamine synthetase; HP: hydroxypyruvate; HTHPP: 2-hydroxyethyl-ThPP; lipoE: lipoamide-E; OSA: oxalosuccinate; NFD: N-formyl-derivatives; 2OG: 2-oxoglutarate; PEP: phosphoenolpyruvate; 3-PGA: 3-phospho-d-glycerate; SADHP-E: S-acetyldihydrolipoamide-E; SLipoE: S-succinyldihydrolipoamide-E; S-7-P: D-sedoheptulose-7P; UCG: UDP-glucuronate decarboxylase; UGDH: UDP-glucose 6-dehydrogenase.

Many DEGs involved in starch and sucrose metabolism, pentose phosphate pathway (PPP), glycolysis/gluconeogenesis, citrate (tricarboxylic acid, TCA) cycle, glyoxylate and dicarboxylate metabolism, pyruvate metabolism, fructose and mannose metabolism, galatose metabolism, and pentose and glucuronate interconversions were identified in J1Cd2 vs J1Cd0 and/or J1Cd5 vs J1Cd0, but far less in J77Cd2 vs J77Cd0 and/or J77Cd5 vs J77Cd0 (Table 1 and Fig 4). Thus, carbohydrate metabolism might be involved in the responses of *A*. *brasiliensis* mycelia to Cd. Trehalose metabolism and PPP can help cells survive under cytotoxic stress, including Cd-toxicity, and that some stresses can route more carbohydrate flux to the two pathways [54]. PPP can provide NADPH for the regeneration of GSH and ASC, thus scavenging ROS [55], and trehalose is a PPP-related stress defender in organisms. In addition to acting as an energy and carbon reserve, trehalose can protect proteins and membranes from denaturation caused by stresses [56]. Guo et al. [54] observed that almost all enzymes involved in trehalose metabolism and PPP increased in Cd-treated cells, and thatincreases in protein abundances correlated with the transcriptional induction. They concluded that growth analysis showed that trehalose metabolism and PPP played a key role in the Cd-tolerance of yeast cells. In this work, they concluded that the regulation of carbohydrate metabolic flux to the two pathways might be a conserved mechanism of dealing with Cd-induced oxidative stress. In this work, we identified three upregulated (viz. *SPAC22F8*.*05*, *fuy1* and *tpp1*) and one downregulated (viz. *YD8119*.*07C*) genes involved in trehalose biosynthesis and degradation, respectively, in J1Cd2 vs J1Cd0 and/or J1Cd5 vs J1Cd0, and one upregulated *fuy1* and one downregulated *YD8119*.*07C* in J77Cd2 vs J77Cd0 and/or J77Cd5 vs J77Cd0. Our results indicated that trehalose level might be elevated in Cd-treated J1 and J77 mycelia, especially in the former due to increased biosynthesis and decreased degradation. Similarly, we isolated two and three upregulated genes involved in PPP in Cd-treated J1 (viz. *FBA1* and *tal1*) and J77 (viz. *gpi1*, *SPAC4G9*.*12* and *TKL1*) mycelia, respectively. Thus, we concluded that trehalose metabolism and PPP were involved in the Cd-tolerance of *A*. *brasiliensis* mycelia. The only exception was that *PFK2* involved in PPP and glycolysis/gluconeogenesis was inhibited in Cd2- and Cd5-treated J77 mycelia. The interconversion of fructose-6-phosphate (F6P) and fructose-1,6-bisphosphate (FBP) is catalyzed glycolytically by an ATP-dependent phosphofructokinase and gluconeogenically by a fructose-1,6-bisphosphatase (FBPase), or is catalyzed by a reversible pyrophosphate (PPi)-dependent phosphofructokinase (PPi-PFK). PPi-PFK catalyzed reaction might provide an adaptive pathway in organisms by using PPi in replace of ATP. The downregulation of *PFK2* implied that the flux of carbohydrates *via* PPi-PFK might be increased, thus enhancing the Cd-tolerance of J77 mycelia.

Gene involved in glycogen biosynthesis (*GLC3*), glycosaminoglycan biosynthesis (*UGDH* and *XUS*), and glycogen biosynthetic and catabolic processes (*GDB1*) were induced in J1Cd2 vs J1Cd0 and J1Cd5 vs J1Cd0, while genes involved in carbohydrate (polysaccharide) catabolic process [viz. *LKA1*, *glaA*, *bglH*, *bglL* (c32133.graph_c0), *agdC* and *bxlB*] were repressed in J1Cd2 vs J1Cd0 and/or J1Cd5 vs J1Cd0. The exceptions were that *bglL* (c31940.graph_c0) and *β-glucosidase 1b* (c31777.graph_c0) involved in polysaccharide catabolic process were induced in Cd-treated J1 mycelia. By contrast, *gpi1* involved in gluconeogenesis and glycolytic process, *GDB1*, *agdC* and *β-glucosidase 1B* (c28258.graph_c0) were induced in J77Cd2 vs J77Cd0 and/or J77Cd5 vs J77Cd0, and that *bglL* (c32133.graph_c0) was inhibited in J77Cd2 vs J77Cd0 and J77Cd5 vs J77Cd0. Thus, the levels of polysaccharides might be enhanced in Cd-treated J1 mycelia, but less in Cd-treated J77 mycelia. This agreed with the more increased requirement for the chelation of Cd in cell wall polysaccharides in Cd-treated J1 mycelia than that in Cd-treated J77 mycelia.

The requirement for energy (ATP) may increase due to initiation of adaptation mechanisms when organisms exposed to Cd-toxicity [57]. The increased energy utilization for ATP-mediated Cd sequestration and export, and biosynthesis of S-containing compounds might be more in Cd-treated J1 mycelia than that in Cd-treated J77 mycelia, because more Cd needed to be sequestrated and exported in the former. Thus, the upregulation of genes involved in energy metabolism might be greater in Cd-treated J1 and J77 mycelia. As expected, we identified far more upregulated DEGs involved in ATP generation (glycolysis/gluconeogenesis, TCA cycle, glyoxylate and dicarboxylate metabolism and pyruvate metabolism) in Cd-treated J1 mycelia than those in Cd-treated J77 mycelia (Fig 4 and Table 1). Therefore, Cd-induced upregulation of energy metabolism might be an adaptive response to meet increased demand for ATP.

Production and secretion of organic acids (OAs) and subsequent formation of relatively immobile heavy metal salts or chelates is an active defense mechanism that fungi mitigate the damage caused by excess heavy metals including Cd [58, 59]. Here, we identified 24 DEGs (22 upregulated and two downregulated genes) related to TCA cycle and glyoxylate and dicarboxylate metabolism, two main pathways involved in OA biosynthesis in Cd-treated J1 mycelia, but not related DEG was observed in Cd-treated J77 mycelia. Therefore, OA biosynthesis might be enhanced in Cd-treated J1 mycelia, thus increasing the secretion of OAs and the immobilization of Cd. This agreed with the more increased requirement for the immobilization of Cd in Cd-treated J1 mycelia.

### DEGs related to cell cycle, DNA replication and repair

Cd is very deleterious to various cellular processes including cell-cycle, and DNA replication and repair. Several *S*. *pombe* mutants involved in cell cycle were sensitive to Cd-toxicity [41, 54]. Here, we isolated four downregulated [viz. *apc2*, *DAM1*, *mad2* and *CDC4* (c27963.graph_c0)] and nine upregulated [viz. *CDC4* (c29933.graph_c0), *slp1*, *cnd1*, *cnd2*, *cnd3*, *rco-1*, *cut1*, *TSD2* and *mph1*) genes involved in cell cycle in J1Cd2 vs Cd0 and/or J1Cd5 vs J1Cd0, but only one downregulated (viz. *mph1*) and two upregulated (viz. *slp1* and *cnd2*) genes in J77Cd5 vs J77Cd0. Also, the expression levels of *slp1*, *cnd1* and *mph1* were higher in J77 mycelia than those in J1 mycelia without Cd (Table 1). Obviously, cell cycle displayed more stability to Cd toxicity in J77 mycelia than that in J1 mycelia, which might contribute to the higher Cd tolerance of J77 mycelia.

Cd can repress all the three major DNA repair pathways (viz. mismatch repair, nucleotide excision repair, and base excision repair) [60, 61]. For several fungi, such as *Exophiala pisciphila* and yeast, positive modulation of DNA repair pathway can restore Cd-induced damage, thus conferring Cd-tolerance [19, 60]. DNA replication as well as DNA repair, is a target of Cd-toxicity. Cd-induced oxidative damage decreased DNA replication but increased repair DNA synthesis during the cell cycle [61]. Yeast mutants lacking *RAD27* encoding Flap exo-endonuclease or *DNAA2* encoding DNA replication ATP-dependent helicase/nuclease DNA2, two enzymes involved in DNA replication and repair, were Cd-hypersensitive [60]. In this study, we identified different genes that are commonly involved in DNA replication and repair in J1Cd2 vs J1Cd0 and J1Cd5 vs J1Cd0, respectively. These includes one downregulated (viz. *RFC2*), six upregulated (viz. ssb1, c30247.graph_c0, POL2, c28697.graph_c0, myh1 and rhp42), two downregulated (viz. c29386.graph_c0 and RFC2) and six upregulated (viz. *ssb1*, c30247.graph_c0, *POL2*, c28697.graph_c0, *myh1* and *rad15*). By contrast, we identified one upregulated *rad15* in J77Cd2 vs J77Cd0, and one downregulated (viz. *apn2*) and ten upregulated (viz. c29386.graph_c0, 24853.graph_c0, rfc3, c30247.graph_c0, *rpb7*, c31675.graph_c0, c19089.graph_c0, c28239.graph_c0, *rad15* and *rnh1*) genes involved in DNA replication and repair in J77Cd5 vs J77Cd0. In addition, the expression levels of four DEGs (viz. c29386.graph_c0, c30247.graph_c0, *POL2* and c31675.graph_c0) were higher in J77 mycelia than those in J1 mycelia at Cd0, but the reverse was the case for the expression level of *RFC2* (Table 1). Obviously, DNA replication and repair pathway displayed more stability in J77 mycelia than in J1 mycelia at Cd2 and greater positive modifications at Cd5. In summary, our results indicated that cell cycle and DNA replication and repair might play a role in Cd-toxicity and Cd-tolerance of *A*. *brasiliensis* mycelia.

## Conclusions

Cd-induced upregulation of *ZIP* might contribute to the higher Cd accumulation in Cd-treated J1 mycelia. Cd might impair cell wall, cell cycle, DNA replication and repair, thus inhibiting J1 mycelium growth. J1 mycelia displayed enhanced formation of S-containing compounds, polysaccharides, OAs, trehalose, ATP and NADPH, and sequestration of Cd to deal with the increased Cd accumulation. DNA replication and repair had better stability at Cd2 treatments; but greater positive modifications at Cd5 treatments; better DNA replication and repair as well as better cell wall and cell cycle stability might contribute to the higher Cd-tolerance of J77 mycelia. Our findings provide a comprehensive set of DEGs influenced by Cd stress; and shed light on molecular mechanism of *A*.*brasiliensis* Cd accumulation and Cd tolerance.

## Supporting information

S1 Fig Gene Ontology (GO) classifications for assembled unigenes of *A*. *brasiliensis* transcriptome. (DOCX)

S2 Fig Histogram presentation of eukaryotic ortholog groups (KOG) classifications for assembled unigenes of *A*. *brasiliensis* transcriptome. (DOCX)

S3 Fig Correlation between qRT-PCR and RNA-Seq results. Points represent average values of three replicates.
(DOCX)

S1 Table The specific primer sequences for qRT-PCR analysis. (DOCX)

S2 Table Summary of the RNA-Seq data collected from control and Cd-treated mycelia of two *A*. *brasiliensis* strains. (DOCX)

S3 Table Length distribution of assembled transcripts and unigenes from two *A*. *brasiliensis* strains. (DOCX)

S4 Table Summary of the functional annotation of assemble unigenes in *A*. *brasiliensis* mycelia. (DOCX)

S5 Table DEGs were identified simultaneously in J1Cd2 vs J1Cd0, J1Cd5 vs J1Cd0, J77Cd0 vs J1Cd0, J77Cd2 vs J77Cd0 and J77Cd5 vs J77Cd0. (DOCX)

S6 Table Significantly enriched KEGG pathway of DEGs from different groups. (DOCX)
