# Supplementary material for: Comparative transcriptome analysis reveals candidate genes related to cadmium accumulation and tolerance in two almond mushroom (Agaricus brasiliensis) strains with contrasting cadmium tolerance
Source: PLoS One. 2020 Sep 29;15(9):e0239617. doi: 10.1371/journal.pone.0239617 (PMC7523953; doi:10.1371/journal.pone.0239617)
Supplement: S1 Fig — (DOCX) [file pone.0239617.s001.docx]

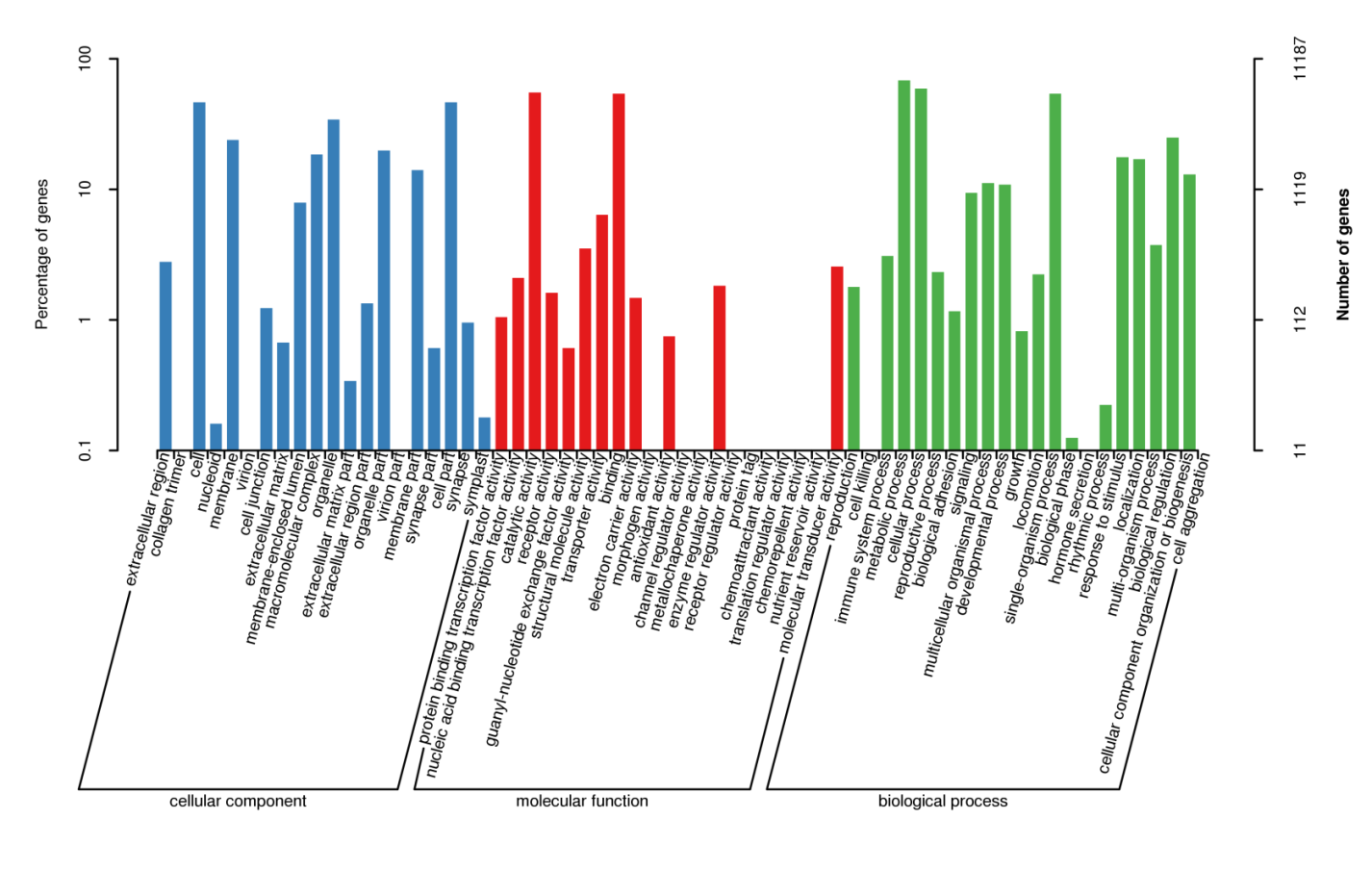
**S1 Fig:** Gene Ontology (GO) classifications for assembled unigenes of *A. brasiliensis* transcriptome. The x-axis represents Go term; the y-axis denotes the number and percentage of unigenes.
