# Supplementary material for: Comparative transcriptome analysis reveals candidate genes related to cadmium accumulation and tolerance in two almond mushroom (Agaricus brasiliensis) strains with contrasting cadmium tolerance
Source: PLoS One. 2020 Sep 29;15(9):e0239617. doi: 10.1371/journal.pone.0239617 (PMC7523953; doi:10.1371/journal.pone.0239617)

**S2 Fig:** Histogram presentation of euKaryotic Ortholog Groups (KOG) classifications for assembled unigenes of *A. brasiliensis* transcriptome. The capital letters in x-axis show the KOG categories as listed on the right, and the y-axis indicates the number of unigenes in each category.
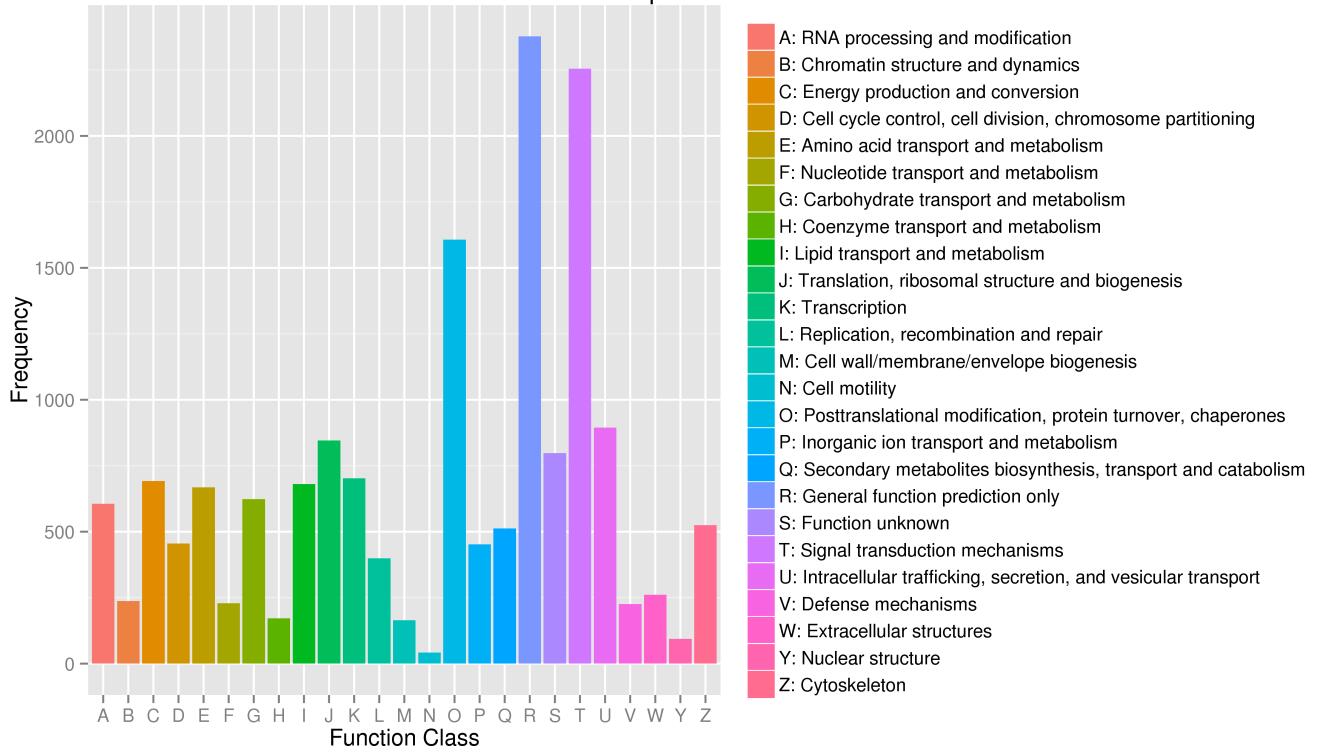

Supplement: S2 Fig — (DOCX) [file pone.0239617.s002.docx]
