# Supplementary material for: Comparative transcriptome analysis reveals candidate genes related to cadmium accumulation and tolerance in two almond mushroom (Agaricus brasiliensis) strains with contrasting cadmium tolerance
Source: PLoS One. 2020 Sep 29;15(9):e0239617. doi: 10.1371/journal.pone.0239617 (PMC7523953; doi:10.1371/journal.pone.0239617)
Supplement: S3 Fig — Points represent average values of three replicates. (DOCX) [file pone.0239617.s003.docx]

**S3 Fig:** Correlation between qRT-PCR and RNA-Seq results. Points represent means of three replicates.
