# Supplementary material for: Comparative transcriptome analysis reveals candidate genes related to cadmium accumulation and tolerance in two almond mushroom (Agaricus brasiliensis) strains with contrasting cadmium tolerance
Source: PLoS One. 2020 Sep 29;15(9):e0239617. doi: 10.1371/journal.pone.0239617 (PMC7523953; doi:10.1371/journal.pone.0239617)
Supplement: S1 Table — (DOCX) [file pone.0239617.s004.docx]

**S1 Table: The specific primer sequences for qRT-PCR analysis**

| Number | Unigene ID | Gene annotation | Gene name | Forward primer sequences (5’-3’) | Reverse primer sequences (5’-3’) |
| --- | --- | --- | --- | --- | --- |
| ***J77Cd2 vs J77Cd0*** | |  |  |  |  |
| 1 | c26465.graph_c0 | Peroxisomal long-chain fatty acid import protein 1 | PXA2 | ACGACGCCACCATAAACAGG | AGCGAGCACGGAAGCAAAC |
| 2 | c26466.graph_c0 | None |  | GATTAGGCTTAGGTGGCAGAGTC | CCAGGCGAGTTTCTTTGGAT |
| 3 | c29937.graph_c1 | Leptomycin B resistance protein pmd1 | pmd1 | CGACATCCTTCTCGCTATCCTC | CGGCTTCACTCAACCATAACG |
| 4 | c32645.graph_c0 | Phosphoadenosine phosphosulfate reductase | sA | ACCTATCATTCCGCACTCTACC | GGAGAACTTCCATTCGACCCTAT |
| 5 | c31374.graph_c0 | Cystathionine gamma-synthase | met-7 | ACAACGATATCTGCATATGGCAAG | GTCTGTTTCGGGTTTCCGTACAC |
| 6 | c29045.graph_c0 | Probable 3-mercaptopyruvate sulfurtransferase | tum1 | CCTAACGCTCCTTCGCCATAC | TCTGATGGTGAATAGGATATCTGG |
| 7 | c27433.graph_c0 | Lanosterol synthase | ERG7 | GATACCTATTCAATCGGGCTCAC | AAGCCAAAGCTCTGGTGGAAC |
| ***J77Cd5 vs J77Cd0*** | |  |  |  |  |
| 8 | c30764.graph_c0 | Acetylornithine aminotransferase, mitochondrial (Precursor) | ARG8 | CTGCTGCCGCTGAAGTTCTAC | CACTTCGCGTTGAGTCGATGT |
| 9 | c26465.graph_c0 | Peroxisomal long-chain fatty acid import protein 1 | PXA2 | ACGACGCCACCATAAACAGG | AGCGAGCACGGAAGCAAAC |
| 10 | c26466.graph_c0 | None |  | GATTAGGCTTAGGTGGCAGAGTC | CCAGGCGAGTTTCTTTGGAT |
| 11 | c30857.graph_c0 | ABC transporter [Iron-sulfur clusters transporter ATM1, mitochondrial (Precursor)] | ATM1 | CTCGTCATCGGTCGCAGTC | ACAATCCTTCCTCCGGCAC |
| 12 | c32822.graph_c0 | Leptomycin B resistance protein pmd1 | pmd1 | CACTTGTTTGCAGCGGTTTC | CCGTGCTCCGATCTTCCCT |
| 13 | c32645.graph_c0 | Phosphoadenosine phosphosulfate reductase | sA | ACCTATCATTCCGCACTCTACC | GGAGAACTTCCATTCGACCCTAT |
| 14 | c31374.graph_c0 | Cystathionine gamma-synthase | met-7 | ACAACGATATCTGCATATGGCAAG | GTCTGTTTCGGGTTTCCGTACAC |
| ***J1Cd2 vs J1Cd0*** | |  |  |  |  |
| 15 | c29285.graph_c0 | Cytochrome P450 61 | SPAC19A8.04 | AGAGAGCCTGCGTGTCAAACC | TGAGCATACTCCAGTCCAATACAT |
| 16 | c18570.graph_c0 | Probable proteasome subunit beta type-3 | pup3 | AAGGAAGAGCGTGAGATTGAAC | ATCGTCTCGAAAAGGTCCTCTG |
| 17 | c30137.graph_c0 | Proteasome subunit alpha type-3 | PRE9 | CTGGCGGATACGGTGGTAGTG | CCGGCGTAAAGGATCGAAAC |
| 18 | c30996.graph_c0 | 26S protease regulatory subunit 6B homolog | tbpA | TCCCATCATCACACCACCATC | TCCCATCATCACACCACCATC |
| 19 | c27072.graph_c0 | C-8 sterol isomerase | ERG2 | AACCGAACGGCAACATCAAC | TGGTACACCATTGGGTACTGAAG |
| 20 | c13031.graph_c0 | Hypothetical protein AGABI2DRAFT_192247 |  | CTCCCTACCTATACCCGACTGC | CTCCGTTATGACCGAGAATGTG |
| 21 | c19606.graph_c0 | Putative aldehyde dehydrogenase-like protein C922.07c | SPAC922.07c | CTCCCTACCTATACCCGACTGC | CTCCGTTATGACCGAGAATGTG |
| 22 | c29993.graph_c0 | Leptomycin B resistance protein pmd1 | pmd1 | CTTTGAACCGTTTGATGCCACT | AGACCTCAATCCACGTCTTTCG |
| 23 | c29411.graph_c0 | 26S proteasome regulatory subunit rpn5 | rpn5b | GAAAGATAAGGCGACGGCAG | CAGGCAAGAGTGAAAGCAGC |
| ***J1Cd5 vs J1Cd0*** | |  |  |  |  |
| 24 | c31374.graph_c0 | Cystathionine gamma-synthase | met-7 | ACAACGATATCTGCATATGGCAAG | GTCTGTTTCGGGTTTCCGTACAC |
| 25 | c27433.graph_c0 | Lanosterol synthase | ERG7 | GATACCTATTCAATCGGGCTCAC | AAGCCAAAGCTCTGGTGGAAC |
| 26 | c29285.graph_c0 | Cytochrome P450 61 | SPAC19A8.04 | AGAGAGCCTGCGTGTCAAACC | TGAGCATACTCCAGTCCAATACAT |
| 27 | c27072.graph_c0 | C-8 sterol isomerase | ERG2 | AACCGAACGGCAACATCAAC | TGGTACACCATTGGGTACTGAAG |
| 28 | c25130.graph_c0 | Metallothionein 2 |  | CTTTATTCATCAATCGAC | CACTAGTCCCCGCGCTGT |
| ***Internal control gene*** | |  |  |  |  |
| 29 | c31752.graph_c1 | Glyceraldehyde-3-phosphate dehydrogenase |  | TTCGGCTCATTTGAAGGGTG | CACGCCAGTCCTTGTGAGAG |
| DEGs validated in two or more comparisons were marked in bold. | | | |  |  |
