# Supplementary material for: Comparative transcriptome analysis reveals candidate genes related to cadmium accumulation and tolerance in two almond mushroom (Agaricus brasiliensis) strains with contrasting cadmium tolerance
Source: PLoS One. 2020 Sep 29;15(9):e0239617. doi: 10.1371/journal.pone.0239617 (PMC7523953; doi:10.1371/journal.pone.0239617)
Supplement: S2 Table — (DOCX) [file pone.0239617.s005.docx]

| **S2 Table:** Summary of the RNA-Seq data collected from control and Cd-treated mycelia of two *A. brasiliensis* strains | | | | | | | | |
| --- | --- | --- | --- | --- | --- | --- | --- | --- |
| Samples | Treatments | Raw reads | Clean reads | Clean reads in raw reads (%) | GC (%) | N (%) | Q30 (%) |  |
| J85Cd0 | J85Cd0-1 | 23947383 | 23844409 | 99.57 | 50.53 | 0.02 | 90.68 |  |
|  | J85Cd0-2 | 21952774 | 21858377 | 99.48 | 50.31 | 0.02 | 91.02 |  |
|  | J85Cd0-3 | 21037636 | 20947174 | 99.62 | 50.54 | 0.02 | 90.78 |  |
| J85Cd2 | J85Cd2-1 | 26313822 | 26200673 | 99.29 | 50.16 | 0.02 | 90.39 |  |
|  | J85Cd2-2 | 22427717 | 22331278 | 99.3 | 50.4 | 0.02 | 90.14 |  |
|  | J85Cd2-3 | 24784793 | 24678218 | 99.32 | 50.62 | 0.02 | 90.21 |  |
| J85Cd5 | J85Cd5-1 | 24708705 | 24602458 | 99.26 | 50.76 | 0.02 | 89.94 |  |
|  | J85Cd5-2 | 23555958 | 23454667 | 99.26 | 50.8 | 0.02 | 90.19 |  |
|  | J85Cd5-3 | 21236588 | 21145271 | 99.36 | 50.92 | 0.02 | 90.51 |  |
| J77Cd0 | J77Cd0-1 | 24084412 | 23980849 | 99.26 | 50.12 | 0.02 | 89.83 |  |
|  | J77Cd0-2 | 24257663 | 24153355 | 99.35 | 50.41 | 0.02 | 90.3 |  |
|  | J77Cd0-3 | 23603527 | 23502032 | 99.37 | 50.63 | 0.02 | 90.61 |  |
| J77Cd2 | J77Cd2-1 | 25410794 | 25301528 | 99.23 | 54.54 | 0.02 | 90.16 |  |
|  | J77Cd2-2 | 24380677 | 24275840 | 99.23 | 50.3 | 0.02 | 90.41 |  |
|  | J77Cd2-3 | 26759482 | 26644416 | 99.21 | 49.9 | 0.02 | 90.45 |  |
| J77Cd5 | J77Cd5-1 | 23709931 | 23607978 | 99.23 | 50.26 | 0.02 | 90.4 |  |
|  | J77Cd5-2 | 26189706 | 26077090 | 99.18 | 50.55 | 0.02 | 90.55 |  |
|  | J77Cd5-3 | 24516514 | 24411093 | 99.23 | 50.45 | 0.02 | 90.46 |  |
