# Supplementary material for: Comparative transcriptome analysis reveals candidate genes related to cadmium accumulation and tolerance in two almond mushroom (Agaricus brasiliensis) strains with contrasting cadmium tolerance
Source: PLoS One. 2020 Sep 29;15(9):e0239617. doi: 10.1371/journal.pone.0239617 (PMC7523953; doi:10.1371/journal.pone.0239617)
Supplement: S3 Table — (DOCX) [file pone.0239617.s006.docx]

**S3 Table:** Length distribution of assembled transcripts and unigenes from two *A. brasiliensis* strains

| Length range | Number of transcripts (%) | Number of unigenes (%) |
| --- | --- | --- |
| 200-300 | 26,706 (9.48%) | 24,227 (41.85%) |
| 301-500 | 20,478 (7.27%) | 16,908(29.21%) |
| 501-1000 | 17,298 (6.14%) | 8,936 (15.44%) |
| 1001-2000 | 36,454 (12.93%) | 3,609 (6.23%) |
| >2000 | 180,907(64.19%) | 4,204 (7.26%) |
| Total number | 281,843 | 57,884 |
| N50 length | 5,790 | 1,519 |
| Mean length | 3,715 | 734 |
