# Supplementary material for: Comparative transcriptome analysis reveals candidate genes related to cadmium accumulation and tolerance in two almond mushroom (Agaricus brasiliensis) strains with contrasting cadmium tolerance
Source: PLoS One. 2020 Sep 29;15(9):e0239617. doi: 10.1371/journal.pone.0239617 (PMC7523953; doi:10.1371/journal.pone.0239617)
Supplement: S4 Table — (DOCX) [file pone.0239617.s007.docx]

**S4 Table:** Summary of the functional annotation of assemble unigenes in *A. brasiliensis* mycelia

| Anno-Database | Number of unigenes | Percentage (%) |
| --- | --- | --- |
| Annotated in COG | 7,708 | 30.72 |
| Annotated in GO | 11,187 | 44.59 |
| Annotated in KEGG | 6,904 | 27.52 |
| Annotated in KOG | 16,517 | 65.83 |
| Annotated in Pfam | 15,598 | 62.17 |
| Annotated in Swissprot | 8,959 | 35.71 |
| Annotated in eggNOG | 17,491 | 69.71 |
| Annotated in Nr | 24,323 | 96.94 |
| Total unigenes | 25,091 | 100 |
