# Supplementary material for: Comparative transcriptome analysis reveals candidate genes related to cadmium accumulation and tolerance in two almond mushroom (Agaricus brasiliensis) strains with contrasting cadmium tolerance
Source: PLoS One. 2020 Sep 29;15(9):e0239617. doi: 10.1371/journal.pone.0239617 (PMC7523953; doi:10.1371/journal.pone.0239617)
Supplement: S5 Table — (DOCX) [file pone.0239617.s008.docx]

**S5 Table:** DEGs were identified simultaneously in J1Cd2 vs J1Cd0, J1Cd5 vs J1Cd0, J77Cd0 vs J1Cd0, J77Cd2 vs J77Cd0 and J77Cd5 vs J77Cd0

| **Number** | **Unigene ID** | **Gene annotation** | **Gene name** | **Log_2_ of fold change** | | | | |
| --- | --- | --- | --- | --- | --- | --- | --- | --- |
|  |  |  |  | **J1Cd2 vs J1Cd0** | **J1Cd5 vs J1Cd0** | **J77Cd0 vs J1Cd0** | **J77Cd2 vs J77Cd0** | **J77Cd5 vs J77Cd0** |
| 1 | c19606.graph_c2 | Aldehyde dehydrogenase | aldA | 1.85 | 3.29 | 2.58 | 2.55 | 3.68 |
| 2 | c22084.graph_c0 | None |  | 2.35 | 2.27 | 1.28 | 1.53 | 1.48 |
| 3 | c22671.graph_c0 | Hypothetical protein |  | 3.92 | 4.6 | 1.88 | 5.01 | 6.15 |
| 4 | **c23427.graph_c0** | Uncharacterized ABC transporter ATP-binding protein/permease YOL075C | YOL075C | 2.09 | 1.82 | 2.66 | -1.57 | -1.94 |
| 5 | c23509.graph_c1 | D-arabinono-1,4-lactone oxidase | alo1 | 1.13 | 2.05 | 1.46 | 1.72 | 2.3 |
| 6 | c25102.graph_c0 | Hypothetical protein |  | 3.31 | 3.62 | 2.29 | 1.76 | 2.28 |
| 7 | c25130.graph_c0 | Metallothionein 2 |  | 7.1 | 8.06 | 3.46 | 4.76 | 6.97 |
| 8 | c25168.graph_c0 | Hypothetical protein |  | -4.62 | -4.07 | -1.21 | -3.78 | -2.48 |
| 9 | **c26780.graph_c0** | Bifunctional solanapyrone synthase (Precursor) | sol5 | 2.89 | 1.52 | 2.71 | -1.19 | -1.59 |
| 10 | c26798.graph_c0 | Cytochrome c peroxidase, mitochondrial (Precursor) | ccp-1 | 4.8 | 4.96 | 3.65 | 2.3 | 2.36 |
| 11 | c28023.graph_c0 | Ent-kaurene oxidase | CYP503A1 | 3.46 | 3.36 | 3.72 | 1.33 | 1.84 |
| 12 | c28322.graph_c0 | Cytochrome P450 52A3-B | CYP52A3-B | 1.75 | 2.76 | 4 | 1.79 | 3.34 |
| 13 | c28519.graph_c0 | Hypothetical protein |  | -6.9 | -7.36 | -1.99 | -2.8 | -3.83 |
| 14 | c28609.graph_c0 | Hypothetical protein |  | 1.62 | 2.65 | 1.97 | 3.65 | 4.69 |
| 15 | c29038.graph_c0 | 1,2-dihydroxy-3-keto-5-methylthiopentene dioxygenase 1 |  | 1.64 | 2.77 | 1.4 | 1.49 | 2.34 |
| 16 | c29206.graph_c0 | BRCT-containing protein 1 | brc1 | -2.55 | -3.31 | -1.29 | -1.41 | -1.58 |
| 17 | c29232.graph_c0 | Carbohydrate-binding module family 13 protein/putative endo-1,3-beta-glucanase [*Agaricus bisporus*] |  | 5.02 | 4 | 5.04 | 1.82 | 1.2 |
| 18 | c29572.graph_c0 | Uncharacterized membrane protein YCR023C | YCR023C | -1.47 | -2.54 | 1.6 | -2.91 | -3.41 |
| 19 | c29646.graph_c0 | 50S ribosome-binding GTPase;; AIG1 family |  | 2.04 | 2.68 | 1.53 | 1.62 | 1.22 |
| 20 | **c30000.graph_c0** | Glucoamylase (Precursor) | glaA | -2.11 | -1.9 | -1.24 | 3.35 | 2.97 |
| 21 | c30397.graph_c0 | Meiotically up-regulated gene 158 protein | mug158 | 1.02 | 1.48 | 1.54 | 1.57 | 2.29 |
| 22 | c31186.graph_c0 | Drug resistance protein YOR378W | YOR378W | 3.47 | 5.2 | 1.94 | 3.36 | 4.82 |
| 23 | **c31241.graph_c0** | Probable alpha/beta-glucosidase agdC (Precursor) | agdC | -2.07 | -1.76 | -1.05 | 2.21 | 3.33 |
| 24 | c31274.graph_c2 | Elongation factor 3 | tef3 | 2.63 | 3.59 | 1.25 | 1.8 | 1.75 |
| 25 | **c31288.graph_c0** | Isotrichodermin C-15 hydroxylase | TRI11 | 1.96 | 1.35 | 3.12 | -2.6 | -2.52 |
| 26 | c31344.graph_c0 | O-methylsterigmatocystin oxidoreductase | ordA | -3.41 | -2.77 | -1.03 | -2.64 | -3.02 |
| 27 | **c31475.graph_c0** | Uncharacterized transporter C1348.05 |  | 2.68 | 1.38 | 3.4 | -2.02 | -2.22 |
| 28 | **c31650.graph_c0** | Gamma-thionin family |  | -1.57 | -1.9 | -1.56 | 1.21 | 2.09 |
| 29 | **c32114.graph_c1** | EF-hand domain pair |  | 1.1 | 1.13 | 1.21 | -1.13 | -1 |
| 30 | **c32287.graph_c1** | Repressor ROX1 | ROX1 | 1.7 | 1.96 | 1.38 | -1.11 | -1.31 |
| 31 | **c32470.graph_c0** | probable cardiolipin-specific deacylase, mitochondrial (Precursor) | SPAC6G10.03c | 1.74 | 1.21 | 1.71 | -1.18 | -1.27 |
| 32 | **c32599.graph_c2** | Phosphatidylinositol 3-kinase vps34 | vps34 | 2.66 | 2.24 | 2.83 | -2.54 | -2.75 |
| 33 | **c32822.graph_c0** | Leptomycin B resistance protein pmd1 | pmd1 | 1.52 | 1.89 | 1.32 | -1.08 | -1.31 |
| 34 | c32895.graph_c0 | Lactam utilization protein lamB | lamB | 2.16 | 1.76 | 2.48 | 2.06 | 2.81 |
| 35 | **c32944.graph_c0** | Hypothetical protein |  | -3.05 | -2.72 | -1.38 | 3.16 | 4.45 |

Note: Unigene IDs displayed opposite expression trends in Cd-treated J1 and J77 mycelia were bold fonts.
