# Supplementary material for: Comparative transcriptome analysis reveals candidate genes related to cadmium accumulation and tolerance in two almond mushroom (Agaricus brasiliensis) strains with contrasting cadmium tolerance
Source: PLoS One. 2020 Sep 29;15(9):e0239617. doi: 10.1371/journal.pone.0239617 (PMC7523953; doi:10.1371/journal.pone.0239617)
Supplement: S6 Table — (DOCX) [file pone.0239617.s009.docx]

**S6 Table:** Significantly enriched KEGG pathway of DEGs from different groups

| Number | Pathway | ko ID | DEG in Pathway | All Gene in Pathway | P-value | Corrected P-value | Gene_id | Group |
| --- | --- | --- | --- | --- | --- | --- | --- | --- |
| 1 | Sulfur metabolism | ko00920 | 8 | 27 | 0.000327 | 0.0323754 | c29045.graph_c0;c30323.graph_c0;c30577.graph_c1;c31374.graph_c0;c31522.graph_c1;c32645.graph_c0;c32857.graph_c0;c32865.graph_c0 | J1Cd5 *vs* J1Cd0 |
| 2 | Steroid biosynthesis | ko00100 | 9 | 45 | 6.78E-05 | 0.0059683 | c12903.graph_c0;c27072.graph_c0;c27433.graph_c0;c28928.graph_c0;c29285.graph_c0;c29809.graph_c0;c31920.graph_c0;c32544.graph_c0;c32879.graph_c0 | J77Cd0 *vs* J1Cd0 |
| 3 | beta-Alanine metabolism | ko00410 | 10 | 58 | 0.000101 | 0.0088836 | c19606.graph_c0;c19606.graph_c2;c19606.graph_c3;c26805.graph_c0;c28325.graph_c0;c29615.graph_c1;c30160.graph_c0;c30540.graph_c0;c30764.graph_c0;c32577.graph_c0 |  |
| 4 | Ribosome | ko03010 | 13 | 276 | 1.93E-05 | 0.0011219 | c13536.graph_c0;c14482.graph_c0;c18594.graph_c0;c22501.graph_c0;c22707.graph_c0;c24014.graph_c0;c26510.graph_c0;c27015.graph_c0;c27847.graph_c1;c29021.graph_c0;c29212.graph_c0;c29405.graph_c0;c31542.graph_c1 | J77Cd2 *vs* J77Cd0 |
| 5 | beta-Alanine metabolism | ko00410 | 6 | 58 | 6.49E-05 | 0.0037636 | c19606.graph_c2;c19606.graph_c3;c29558.graph_c0;c30540.graph_c0;c30764.graph_c0;c32143.graph_c1 |  |
| 6 | Galactose metabolism | ko00052 | 5 | 44 | 0.000177 | 0.010251 | c26976.graph_c0;c30966.graph_c0;c31241.graph_c0;c32050.graph_c1;c32902.graph_c0 |  |
| 7 | beta-Alanine metabolism | ko00410 | 11 | 58 | 7.11E-06 | 0.000597 | c19606.graph_c0;c19606.graph_c1;c19606.graph_c2;c19606.graph_c3;c26805.graph_c0;c27385.graph_c0;c28325.graph_c0;c29558.graph_c0;c29805.graph_c0;c30540.graph_c0;c30764.graph_c0 | J77Cd5 *vs* J77Cd0 |
| 8 | Histidine metabolism | ko00340 | 8 | 40 | 8.95E-05 | 0.0075143 | c19606.graph_c0;c19606.graph_c1;c19606.graph_c2;c19606.graph_c3;c26805.graph_c0;c27866.graph_c0;c28325.graph_c0;c29558.graph_c0 |  |
| 9 | Glycerolipid metabolism | ko00561 | 10 | 66 | 0.000141 | 0.0118098 | c19606.graph_c0;c19606.graph_c1;c19606.graph_c2;c19606.graph_c3;c26805.graph_c0;c26976.graph_c0;c28325.graph_c0;c28846.graph_c0;c29558.graph_c0;c31270.graph_c0 |  |
| 10 | Ascorbate and aldarate metabolism | ko00053 | 7 | 36 | 0.000301 | 0.0252988 | c19606.graph_c0;c19606.graph_c1;c19606.graph_c2;c19606.graph_c3;c26805.graph_c0;c28325.graph_c0;c29558.graph_c0 |  |
| 11 | Pentose and glucuronate interconversions | ko00040 | 8 | 50 | 0.000454 | 0.038135 | c19606.graph_c0;c19606.graph_c1;c19606.graph_c2;c19606.graph_c3;c26805.graph_c0;c28325.graph_c0;c29558.graph_c0;c30966.graph_c0 |  |
